# Immediate Dosage Compensation Is Triggered by the Deletion of Y-Linked Genes in *Silene latifolia*

**DOI:** 10.1016/j.cub.2019.05.060

**Published:** 2019-07-08

**Authors:** Marc Krasovec, Yusuke Kazama, Kotaro Ishii, Tomoko Abe, Dmitry A. Filatov

**Affiliations:** 1 Department of Plant Sciences, University of Oxford, Oxford OX1 3RB, UK; 2 RIKEN Nishina Center for Accelerator-Based Sciences, 2-1 Hirosawa, Wako, Saitama, 351-0198, Japan

**Keywords:** sex chromosome, dosage compensation, *Silene latifolia*, Y deletion, white campion

## Abstract

The loss of functional genes from non-recombining sex-specific chromosomes [1, 2], such as the Y chromosomes in mammals [3] or W chromosomes in birds [4], should result in an imbalance of gene products for sex-linked genes [5]. Different chromosome-wide systems that rebalance gene expression are known to operate in organisms with relatively old sex chromosomes [6]; e.g., *Drosophila* overexpress X-linked genes in males [7], while mammals shut down one of the X chromosomes in females [8]. It is not known how long it takes for a chromosome-wide dosage compensation system to evolve. To shed light on the early evolution of dosage compensation, we constructed a high-density Y-deletion map and used deletion mutants to manipulate gene dose and analyze gene expression in white campion (*Silene latifolia*), which evolved dioecy and sex chromosomes only 11 million years ago [9]. We demonstrate that immediate dosage compensation can be triggered by deletions in a large portion of the p arm of the Y chromosome. Our results indicate that dosage compensation in *S. latifolia* does not have to evolve gene by gene because a system to upregulate gene expression is already operating on part of the X chromosome, which likely represents an intermediate step in the evolution of a chromosome-wide dosage compensation system in this species.

## Highlights

• Dosage compensation on *Silene latifolia* X chromosome can be triggered by Y deletions
• We constructed a new high-density deletion map of *S. latifolia* Y chromosome
• The gene(s) controlling X upregulation map to a region on the p arm of the Y chromosome
• DNA methylation and imprinting are not involved in *S. latifolia* dosage compensation

## Teaser

Krasovec et al. use Y deletions to study evolution of dosage compensation on recently evolved sex chromosomes in *Silene latifolia*. They report that experimental deletion of Y-linked genes leads to immediate upregulation of X gametologs, indicating that dosage compensation does not have to evolve gene by gene with progressing Y degeneration.

## Results and Discussion

Very little is known about dosage compensation (DC) in plants [10, 11, 12, 13]. The *de novo* evolution of sex chromosomes in *Silene latifolia* (*Caryophyllaceae*) only 11 million years ago [9] offers a rare opportunity to analyze the early stages of sex chromosome evolution, with actively progressing Y degeneration and evolving DC “caught in action.” The previous studies on *S. latifolia* [12, 13] reported signs of a nascent DC system upregulating X-linked genes with degenerate or partially degenerate Y-linked gametologs—the homologous genes shared between the X and the non-recombining Y chromosomes. As *S. latifolia* is not easily amenable to transgenic analyses, the molecular bases of this DC remain unexplored. In particular, it remains unclear whether DC has to evolve gene by gene—independently at each sex-linked gene that lost its Y-linked gametolog or if some form of chromosome- or region-wide DC is already present on *S. latifolia* sex chromosomes. Here, we make use of a set of Y-deletion mutants generated in an *S. latifolia* inbred strain [14] to test whether experimental deletion of the Y-linked genes leads to altered expression of their respective X gametologs. If DC in *S. latifolia* is required to evolve gene by gene, then Y-linked gene deletion should not immediately lead to altered expression of the corresponding X gametolog, as there would not have been time for it to evolve. On the other hand, if immediate dosage compensation (IDC) is observable for X gametologs of experimentally deleted Y-linked genes, it would imply that some form of DC is already operating.

### Y Deletion Mutants and the Deletion Map of *S. latifolia* Y Chromosome

In this study, we used 101 *S. latifolia* Y-deletion mutants (Table S1), 67 of which have not been described previously. All these mutants were generated by heavy ion irradiation of pollen grains or dry seeds [14]. As the original aim of generating these mutants was to precisely locate the sex-determining gynoecium-supressing function (GSF) and stamen-promoting function (SPF) genes on the Y chromosome, most of these mutants were initially identified by morphological changes in male or female organs in the flowers [14]. As Y-deletion mutants contain Y chromosomes, they are genetically males, but deletions of sex-determining SPF and/or GSF genes altered the presence of stamens and pistils in the flower (Table S1). The presence of Y deletions in each mutant was analyzed by PCR-based genotyping of 163 STS markers (Table S2), with 0 to 81 markers found to be deleted per mutant. The presence of deletions in other chromosomes was analyzed in EGP14, EGP17, and mut3-98 mutants relative to control, for which approximately 80 Gb of sequence data with 150 bp paired-end reads were generated per individual. No deletions outside the Y chromosome were found except one autosomal deletion in linkage group 4 of mutant mut3-98 (see below).

Following the previously described approach [14], we constructed a new high-density deletion map for the *S. latifolia* Y chromosome (Table S1), which is a significant improvement over the previous iteration of a Y-deletion map that was based on only 41 mutants and 71 Y-linked markers [14]. The new map allowed us to locate the Y deletions affecting expression of X-linked genes (see below) as well as narrow down the regions in which the sex-determining genes are located (Table S1). Due to pre-selection of the mutants by morphological changes in male of female organs in the flowers [14], most Y deletions clustered around the two sex-determining genes in the p arm, although a few deletions were also found in the q arm of the Y chromosome (Table S1).

Seventeen of the Y-deletion mutants and three controls plants that were still actively growing at the time of the study were used for high-throughput transcriptome sequencing (Data S1). Using sequence coverage in the sequenced mutant males and non-irradiated controls, we reconfirmed the Y deletions identified previously [14]. As expected, the deleted Y gametologs had zero sequence coverage in the deletion mutants, and active expression was confirmed in the control plants (Data S1). Using this approach, combined with PCR-based verification, we identified 177 Y-linked genes deleted in the irradiated mutants, including 35 such genes identified previously [14]; 127 of these genes were independently deleted in at least two Y-deletion mutants. All of these Y-linked genes have actively expressed X-linked gametologs whose expression was analyzed in controls and deletion mutants (Data S1).

### Expression of X-Linked Genes in Y-Deletion Mutants

To study gene expression changes caused by deletion of Y-linked genes, we analyzed transcriptome sequence data for leaves and flower buds of the deletion mutants and controls with up to three technical replicates per sample (Data S1). Gene expression, measured as FPKM (fragments per kilobase per million [15]) values, showed a high correlation between technical replicates (Pearson’s ρ ≥ 0.82; p < 2.2 × 10^−16^) and between mutants and controls (Pearson’s ρ ≥ 0.86; p < 2.2 × 10^−16^). The latter indicates that the overall expression is very similar between the mutants and controls, so the FPKM normalization across samples is adequate, and no external spike in normalization [16] was necessary, as has been done in some studies of chromosomal aneuploidy [17].

**Figure 1:**
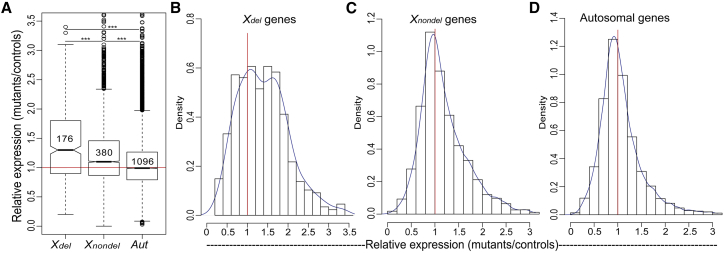
Relative Expression of X-Linked and Autosomal Genes in Leaves of Mutants Compared to Controls The X-linked genes are split into two categories: the genes for which Y-linked gametologs were deleted (*X*_*del*_) and not deleted (*X*_*nondel*_) in the deletion mutants. (A) Significant upregulation of X-linked genes in the mutants compared to controls is evident in both categories of X-linked genes, though it is much stronger in the *X*_*del*_ compared to *X*_*nondel*_ genes. The numbers of genes analyzed in each category are shown within the boxplot. The difference between gene categories was tested for significance with the Wilcoxon rank sum test, ^∗∗∗^p < 0.001. In the box-and-whisker plot (A), the box spans the first quartile (Q1) to the third quartile (Q3); the line inside the box shows the median; the notches represent the 95% confidence interval for the median; and whiskers show the range between Q1 − 1.5 × (Q3 − Q1) and Q3 + 1.5 × (Q3 − Q1). (B–D) Distributions of expression change in the mutants compared to controls for *X*_*del*_ genes (B), *X*_*nondel*_ genes (C), and autosomal genes (D). The curves in (B) to (D) show the kernel-smoothed density function. The red lines at 1 in all panels show the null expectation for no difference in expression between deletion mutants and controls. Data used for this figure are listed in Data S1. See also Figure S1.

In order to compare the effect of the deletion of a Y gene on the expression of the homologous X-linked genes with the effect of the same deletion on the genes for which both copies are present, we split the sex-linked genes into “del” and “nondel” categories (Figure 1). Only the X-linked genes with Y gametolog deleted in the particular mutants were included in the *X*_*del*_ category. Similarly, the *X*_*nondel*_ category included X-linked genes with the Y gametolog non-deleted in the particular mutants. We first focused on the general pattern of gene expression following the deletion, regardless of the degeneration state of the Y gametolog and its location on the chromosome. The *X*_*del*_ genes significantly increased their expression compared to expression of the same genes in the control plants without Y deletions (Figure 1A), and this increase is significantly stronger compared to *X*_*nondel*_ and *Aut* genes (Figure 1A; Wilcoxon rank sum tests, p < 0.001 for all comparisons). This result indicates the presence of some form of IDC in *S. latifolia*. Interestingly, the distribution of *X*_*del*_ expression change in the mutants compared to controls is bimodal, with peaks at around 1 and 1.8 (Figure 1B), which contrasts with unimodal distributions for *X*_*nondel*_ and *Aut* genes (Figures 1C and 1D, respectively). This indicates that a fraction of *X*_*del*_ genes nearly double expression in the mutants, while other *X*_*del*_ genes do not change expression. A weaker but still significant increase in expression was also observed for the *X*_*nondel*_ genes (Figure 1A), indicating that the effect of Y deletions may extend beyond the *X*_*del*_ genes. This contrasted with little change in the deletion mutants compared to control plants for autosomal genes (Figure 1A).

The genes of *S. latifolia* sex chromosomes fall into at least two evolutionary strata—the regions of similar divergence between the X and Y gametologs created by stepwise expansion of the non-recombining region on the Y chromosome [13, 18]. The silent divergence between the X and Y gametologs and the extent of Y degeneration is somewhat higher in the older (∼11 million years [my] [9]) compared to younger (∼6 my [9]) evolutionary stratum [9, 13, 19]. Grouping genes by the extent of synonymous divergence (*dS*) between the X and Y gametologs (Figures S1A–S1G) or by the extent of degeneracy of Y-linked genes (measured by Y_c_/X_c_ expression ratio in controls) deleted in the mutants (Figures S1I–S1O) revealed the presence of IDC for all categories of *X*_*del*_ genes, though upregulation was stronger in non-degenerate Y gametologs (Y_c_/X_c_ > 0.7, Figure S1) and in genes with *dS* > 0.04 (Figure S1). Weaker but still significant upregulation in mutants compared to that in controls was also observed in most categories of *X*_*nondel*_ genes (Figure S1).

### IDC Is Caused by Y Deletions Located in a Region between the SPF and GSF Genes

**Figure 2:**
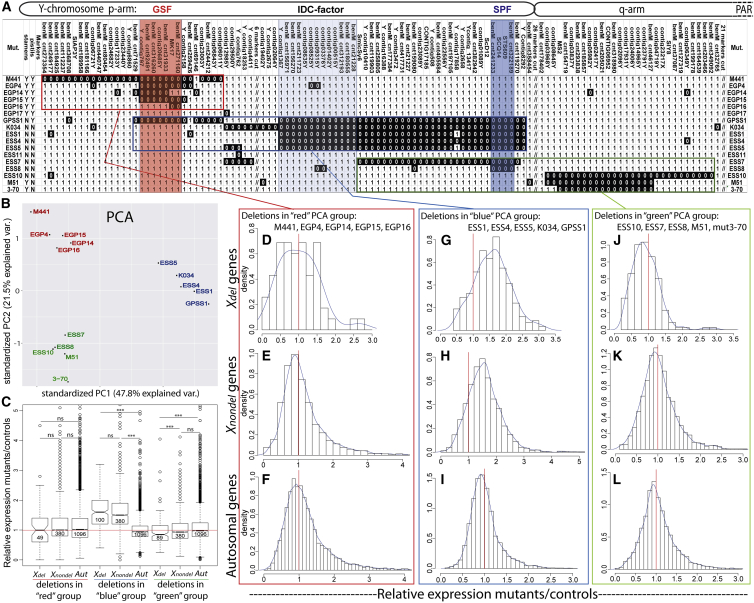
Upregulation of X-Linked Genes Is Caused by Deletions in the IDC-Factor Region of the P Arm of the Y Chromosome (A) Location of Y deletions in the mutants with sequenced transcriptomes (the full map is shown in Table S1). The presence and absence (deletion) of a marker in the particular mutant is indicated with 1 (white background) and 0 (black background), respectively. Note that 53 uninformative Y markers were excluded (marked with “//”) to make this figure narrower. Primers used for genotyping Y markers are listed in Table S2. (B) Clustering of mutants by relative expression in principle components analysis (PCA). (C–L) Boxplot comparing relative expression for the same categories of genes as shown in (D) to (L). The significance for these comparisons was tested with Wilcoxon rank sum test, ^∗∗∗^p < 0.001. The numbers of genes analyzed in each category are shown within the boxes on the boxplot. The box in this box-and-whisker plot (C) spans the first quartile (Q1) to the third quartile (Q3); the line inside the box shows the median; the notches represent the 95% confidence interval for the median; and whiskers show the range between Q1 − 1.5 × (Q3 − Q1) and Q3 + 1.5 × (Q3 − Q1). The red lines in (C) to (L) show the null expectation for no difference in expression between deletion mutants and controls. The blue curves in (D) to (L) show the kernel-smoothed density function for the distributions of relative expression in each category. To control for expression differences that could be caused by deletion of GSF or SPF loci and associated morphology changes, the hermaphroditic EGP mutants were normalized by expression in hermaphroditic mutant EGP17 that contained no big deletions and asexual ESS and K034 mutants were normalized by expression in asexual mutant ESS11 that also lacked big deletions, while q-arm mutants M51 and 3-70 that had a normal male phenotype were normalized by non-irradiated controls. The same analysis with all mutants normalized by non-irradiated controls is shown in Figure S2. Expression data used for these analyses are listed in Data S1. See also Figures S3 and S4 for related analyses testing whether upregulation of X-linked genes is caused by methylation changes or imprinting.

To test whether the location of deletions on the Y chromosome affects IDC, we used our new high-density deletion map of the Y chromosome (Table S1). A subset of the Y-deletion map, including the mutants for which expression was analyzed in the current study, is shown in Figure 2A. Principal components analysis (PCA) of gene expression in the mutants relative to that in controls revealed that mutants form three distinct clusters (hereby referred to as red, blue, and green; Figure 2B) according to the location of Y deletions in the Y chromosome. The analysis of relative expression for each mutant separately (Table 1) revealed the presence of IDC only in the five mutants of the blue PCA cluster (GPSS1, ESS1, ESS4, ESS5, and K034) that have deletions along a large part of the p arm of the Y chromosome. In contrast, deletions elsewhere on the Y chromosome do not appear to trigger IDC (Table 1 and Figure 2). This result is consistent across the tissues analyzed (Table 1).

**Table 1:** Gene Expression in the Mutants Relative to Non-Irradiated Controls

Mutants:	ESS1	ESS4	ESS5	K034	GPSS1	M441	EGP4	EGP14	EGP15	EGP16	EGP17	ESS11	ESS7	ESS8	ESS10	M51	mut3-70
**Leaf Expression in the Mutants Relative to Controls (median ± SD)**																	

Xdel	**1.6 ± 0.64**	**1.6 ± 0.51**	**1.5 ± 0.44**	**1.7 ± 0.42**	**1.8 ± 0.78**	0.7 ± 0.36	0.9 ± 0.42	0.7 ± 0.02	0.9 ± 1.16	N/A	N/A	N/A	0.9 ± 0.31	N/A	0.8 ± 0.32	0.9 ± 0.36	0.8 ± 0.51
Xnondel	**1.7 ± 0.66**	**1.6 ± 0.55**	**1.5 ± 0.61**	**1.6 ± 0.45**	**1.7 ± 0.84**	0.9 ± 0.39	0.9 ± 0.31	1.0 ± 0.32	1.0 ± 0.28	1.0 ± 0.28	1.0 ± 0.45	1.1 ± 0.41	1.0 ± 0.47	0.9 ± 0.35	1.0 ± 0.29	1.0 ± 0.47	1.0 ± 0.54
Ynondel	1.0 ± 0.64	1.0 ± 0.48	0.9 ± 0.47	1.0 ± 0.34	1.0 ± 0.53	0.9 ± 0.45	1.0 ± 0.34	1.0 ± 0.36	1.0 ± 0.30	1.0 ± 0.33	0.9 ± 0.44	1.0 ± 0.42	1.0 ± 0.43	0.9 ± 0.32	1.0 ± 0.40	0.9 ± 0.61	0.9 ± 0.62
Autosomal	1.0 ± 0.43	1.0 ± 0.33	0.9 ± 0.39	0.9 ± 0.28	1.0 ± 0.41	1.0 ± 0.41	0.9 ± 0.37	1.1 ± 0.41	1.0 ± 0.39	1.0 ± 0.38	1.0 ± 0.57	1.0 ± 0.40	1.0 ± 0.39	0.9 ± 0.27	1.0 ± 0.34	1.0 ± 0.81	1.0 ± 0.80

**Number of Genes Analyzed for Leaf Expression: Autosomal = 1,096 Genes Analyzed in Each Mutant**																	

“del”	58	47	50	71	65	43	9	2	4	0	0	0	29	0	60	46	39
“nondel”	322	333	330	309	315	335	371	378	376	380	380	380	351	380	320	334	341

**Bud Expression in the Mutants Relative to Controls (median ± SD)**																	

Xdel	N/A	**1.6 ± 0.56**	**1.6 ± 0.56**	N/A	N/A	0.7 ± 1.39	0.9 ± 0.50	0.6 ± 0.18	1.0 ± 0.63	N/A	N/A	N/A	0.9 ± 0.34	N/A	N/A	N/A	N/A
Xnondel	N/A	**1.6 ± 0.55**	**1.6 ± 0.54**	N/A	N/A	0.9 ± 0.87	1.0 ± 0.31	0.9 ± 0.38	1.0 ± 0.26	1.0 ± 0.24	N/A	N/A	1.0 ± 0.38	N/A	N/A	N/A	N/A
Ynondel	N/A	1.0 ± 0.56	1.0 ± 0.60	N/A	N/A	0.9 ± 0.77	1.0 ± 0.57	0.9 ± 0.55	1.0 ± 0.54	1.0 ± 0.41	N/A	N/A	1.0 ± 0.43	N/A	N/A	N/A	N/A
Autosomal	N/A	0.9 ± 0.51	0.9 ± 0.43	N/A	N/A	0.9 ± 1.89	1.0 ± 0.68	1.0 ± 0.62	1.0 ± 0.39	1.0 ± 0.37	N/A	N/A	1.0 ± 0.42	N/A	N/A	N/A	N/A

**Number of Genes Analyzed for Bud Expression: Autosomal = 1,096 Genes Analyzed in Each Mutant**																	

“del”	N/A	48	51	N/A	N/A	43	9	2	4	0	N/A	N/A	30	N/A	N/A	N/A	N/A
“nondel”	N/A	333	330	N/A	N/A	338	372	379	377	381	N/A	N/A	351	N/A	N/A	N/A	N/A

Gene categories significantly upregulated in the mutants are shown in bold (p < 0.01; Wilcoxon test). Data used for this table are listed in Data S1.

The five mutants of the blue cluster show IDC in both the *X*_*del*_ and *X*_*nondel*_ genes, while Y-linked or autosomal genes show no upregulation compared to controls (Figure 2 and Table 1). This indicates that the mechanism responsible for upregulated X expression acts similarly on *X*_*del*_ and *X*_*nondel*_ genes, but its action is limited to X-linked genes. Using the Y-deletion map, it is possible to locate the factor(s) responsible for triggering X upregulation. The Y region deleted in all five blue cluster mutants, but not in any other mutants, is located proximally to GSF and distally to SPF regions and includes only 13 Y markers, bordered by Ycontig1367 and BenM_cnt21228 (the “IDC-factor” region on Figure 2A). However, it is likely an oversimplification that only deletions in the IDC-factor region trigger IDC, because significant up- or downregulation is also observed for a number of *X*_*del*_, *X*_*nondel*_, and *Aut* genes in the mutants of all three PCA clusters, as revealed by the volcano plots in Figure S2.

### Is IDC Specific to the X Chromosome?

Although dosage compensation systems are usually discussed in the context of sex chromosomes, variation in gene dosage can also be found elsewhere in the genome [20]. For example, partial dosage compensation for gene duplications or deletions has been found on *Drosophila* autosomes [21] and in species that have no sex chromosomes [17, 22, 23]. The role of such compensatory upregulation is not well understood and may partly account for dosage compensation in *S. latifolia* and in other plant and animal species.

**Figure 3:**
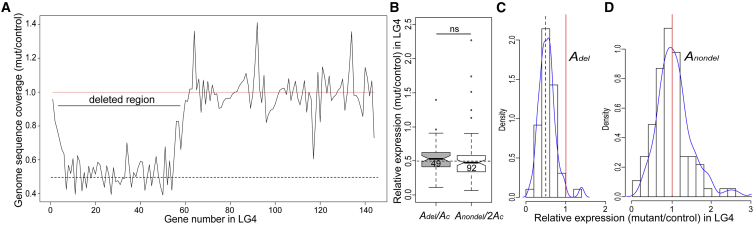
No Evidence for Dosage Compensation in Genes Deleted from Autosomal Linkage Group 4 (LG4) in Leaf Transcriptomes (A) Graph showing genome sequencing coverage for LG4. The genes are ordered according to their position on LG4. (B–D) The autosomal genes within the heterozygous deletion (*A*_*del*_) are expressed at ∼50% of their total expression in controls (*A*_*c*_*)* that are homozygous for no deletion, indicating no dosage compensation in this region. *A*_*nondel*_ denotes autosomal genes on LG4 outside the deletion in the mutant mut3-98. *A*_*nondel*_ expression for each gene was halved, and *A*_*del*_ and *A*_*nondel*_ values were normalized by expression of the same genes in the controls. The numbers of genes analyzed in each category are shown inside the boxes. The box in this box-and-whisker plot (B) spans the first quartile (Q1) to the third quartile (Q3); the line inside the box shows the median; the notches represent the 95% confidence interval for the median; and whiskers show the range between Q1 − 1.5 × (Q3 − Q1) and Q3 + 1.5 − (Q3 − Q1). (C) and (D) show the probability density histograms (with kernel-smoothing function) for *A*_*del*_ and *A*_*nondel*_ genes, respectively. (B), (C), and (D) are based on the same gene expression data in the mutant mut3-98 relative to control. The dashed line at 0.5 in (A), (B), and (C) show the expectation for deleted (hemizygous) region, while the red line at 1 in (A), (C), and (D) shows the expectation for non-deleted region. Data are listed in Table S3.

In order to test whether the IDC can also be triggered on *S. latifolia* autosomes, we analyzed gene expression in an autosomal deletion mutant mut3-98 that was generated via heavy ion irradiation of pollen of an inbred *S. latifolia* strain in the same way as in the Y-deletion mutants described above. To identify the genes deleted in mut3-98, we sequenced its genome with the Illumina HiSeq2500 platform and compared sequence coverage in the mutant and non-irradiated control (Figure 3A and Table S3). This analysis identified 49 autosomal genes that are hemizygous in the mutant as a result of a heterozygous deletion on linkage group 4 (LG4) of the previously published genetic map [13]. Note that only genes present in the map were used in this analysis, as the location of the other genes is unknown. The deleted genes are located in a single contiguous region that is 47.17 cM long and therefore comprises a large portion of LG4 (Figure 3A). The genetic map was used to establish the location of the deleted genes because the genome assembly for *S. latifolia* remains highly fragmented [9, 13]. To test for dosage compensation in the 49 deleted autosomal genes, expression of each hemizygous gene within the heterozygous autosomal deletion (*A*_*del*_) was compared with the expression of the same gene in the controls that were homozygous for no deletion (*A*_*c*_*A*_*c*_). The null expectation for genes with no dosage compensation is *A*_*del*_ ∼*A*_*c*_*A*_*c*_/2, while *A*_*del*_ ∼*A*_*c*_*A*_*c*_ is expected for genes with dosage compensation. The observed *A*_*del*_ expression did not differ significantly from the expectation for no dosage compensation (Wilcoxon test; W = 692, p = 0.44, ratio *A*_*del*_/*A*_*c*_*A*_*c*_ compared to 0.5; Figures 3B–3D), indicating an absence of dosage compensation in this part of the genome and possibly elsewhere outside the sex chromosomes in *S. latifolia*. Furthermore, the distribution of *A*_*del*_ relative expression did not significantly differ for the autosomal genes inside and outside the deleted region in mut3-98 (Wilcoxon test; W = 2,575, p = 0.17; Figure 3B). Thus, unlike the deletions on the Y chromosome, the deletion of autosomal genes does not trigger IDC in this region, indicating that the mechanism(s) responsible for immediate upregulation of gene expression may be active only on the sex chromosomes of *S. latifolia*. However, it is worth noting that the analysis of a single autosomal deletion cannot rule out the presence of IDC on other autosomes. Indeed, the inverse effect of chromosome dosage on gene expression was described for aneuploids in several other animal and plant species [17, 21, 22, 23], which suggests the generality of this phenomenon. Unfortunately, no other autosomal deletions are available for *S. latifolia* to test the presence of IDC in other parts of the genome.

### What Are the Molecular Bases of Dosage Compensation in *S. latifolia*?

We demonstrated that expression of *S. latifolia* X-linked genes can be upregulated by experimental deletion of Y-linked gametologs. Transcriptional buffering [21]—passive attenuation of gene dosage changes due to non-linear properties of the transcription system [24]—is probably the simplest explanation for such IDC. However, the transcriptional buffering hypothesis does not explain why IDC is triggered only by the deletions in the Y chromosome IDC-factor region on Figure 2A. A related mechanism—simultaneous change in gene dose of a negative regulator(s) and the target genes [16, 25, 26, 27, 28]—was proposed to explain dosage compensation in aneuploids in many organisms [29, 30, 31, 32], including in the species without sex chromosomes, such as maize [22] and *Arabidopsis* [17]. If this mechanism is responsible for IDC in *S. latifolia*, the deletions in the IDC-factor region cause IDC because they reduce the dosage of some gene(s) that negatively regulate(s) expression of many X-linked genes, while deletions elsewhere on the Y chromosome do not trigger IDC because they do not affect such negative regulator(s). However, this mechanism does not explain why deletions on the Y chromosome primarily affect X-linked genes, while their *trans* effect on autosomal expression is weak. This suggests that IDC may act via some cis-mechanism on the sex chromosomes, such as alteration of chromatin state to enhance the X chromosome chromatin accessibility [33], not dissimilar to that reported for animal dosage compensation systems [34, 35, 36, 37]. Alternatively, the IDC could work via re-location of the X chromosome inside the nucleus [35, 36, 37], resulting in alteration of the 3D chromatin architecture, as recently reported for plant genomes [38, 39]. Furthermore, these *cis*-acting mechanisms may work in combination with a *trans*-acting negative regulator of expression, as proposed for *Drosophila* [25], that results in two-fold X upregulation and a weaker autosomal effect. Indeed, the volcano plots reveal a small fraction of autosomal genes that are significantly up- or downregulated (Figure S2).

DNA methylation is known to be involved in gene regulation [40] on sex chromosomes and autosomes of many species [41] and was suggested to play a role in sex chromosome evolution in *S. latifolia* [42]. If DNA methylation plays a role in IDC, we expect methylation to differ between the mutants and controls, particularly so at the *X*_*del*_ and *X*_*nondel*_ genes for deletions in the blue PCA cluster mutants. In order to test this, we compared the extent of DNA methylation between ESS1 and ESS4 deletion mutants and control plant using genomic sequencing of bisulphite-treated DNA (25 to 26 Gb of sequence data per plant). Methylation was detected mainly at CpG sites, while only ∼2% of CHH and CHG sites were methylated in the samples analyzed. The analysis of coding regions and the adjacent 5′ untranslated regions revealed no difference in methylation between the mutants and control (Figure S3). Thus, DNA methylation is unlikely to be involved in *S. latifolia* IDC. This result is consistent with the analysis of methylation changes in *A. thaliana* aneuploids, which concluded that “genetic imbalance is generally mechanistically unrelated to DNA methylation” [17].

Based on the analysis of allele-specific expression in females, a recent study reported that dosage compensation in *S. latifolia* acts via maternal imprinting, with the X chromosome inherited from the mother upregulated in both male and female progeny [43]. Such X-chromosome-wide upregulation would result in overcompensation for all X-linked genes in females and for X-linked genes with functional Y gametologs in males. In males, this overcompensation may be ameliorated by Y-linked factor(s) reducing X expression. Deleting these X-suppressing Y-linked factor(s) in Y-deletion mutants may be the cause of IDC detected in our study. Thus, if the *S. latifolia* X chromosome is indeed upregulated, as reported by Muyle et al. [43], the IDC may be a secondary adaptation aimed at preventing overcompensation of sex-linked genes in males. However, re-analyzing published transcriptome sequence data [13], which was obtained independently from the data of [43], we were unable to confirm the conclusion of Muyle et al. [43] that dosage compensation in *S. latifolia* acts via maternal imprinting. Our dataset comprised transcriptome data for parents and 52 progeny (20 males and 32 females) of an *S. latifolia* genetic cross [13]. In our re-analysis of allele-specific expression in an *S. latifolia* genetic cross, the maternal and paternal alleles of X-linked genes in females were equally expressed (Figure S4), which contradicts the results of Muyle et al. [43]. Thus, the role of maternal imprinting in *S. latifolia* dosage compensation has to be taken with caution and requires independent verification. Clearly, the molecular bases of dosage compensation in *S. latifolia* require further analysis.

### Conclusions

Overall, our results indicate the presence of a pre-existing dosage compensation system in some regions of *S. latifolia* sex chromosomes. This system can upregulate X-linked genes immediately after loss of their Y-linked gametologs. Thus, many X-linked genes do not have to evolve dosage compensation in a gene-by-gene fashion as Y-linked gametologs degenerate. The immediate dosage compensation of X-linked genes appears to be triggered by deletions in a part of the p arm of the Y chromosome—the IDC-factor region—that likely contains gene(s) controlling expression of the X chromosome. The finding of IDC on the *S. latifolia* sex chromosomes is surprising, given the recent origin of dioecy and sex chromosomes in this species. IDC described in this study may represent an intermediate step in the evolution of a chromosome-wide dosage compensation system. A scenario of how pre-existing IDC may have played central role in evolution of dosage compensation in *Drosophila* was described by Birchler [25]. Similarly, IDC may be the basis of evolving dosage compensation in *S. latifolia*, although the molecular mechanisms of this process remain to be studied.

## STAR★Methods

### Key Resources Table

**Table:** 

REAGENT OR RESOURCE	SOURCE	IDENTIFIER
**CRITICAL COMMERCIAL ASSAYS**		

Illumina paired end genomic sequencing	WTCHG, UK	Paired end genomic sequencing
Illumina transcriptome sequencing	WTCHG, UK	Paired end sequencing of polyA RNA
Illumina high throughput sequencing of bisulfite-treated genomic DNA	BGI, Hong Kong	Paired end bisulfite sequencing
QIAGEN RNeasy Plant Mini Kit	QIAGEN	cat # 74904
QIAGEN DNeasy Plant Kit	QIAGEN	cat # 69104

**DEPOSITED DATA**		

Genome sequencing data of *S. latifolia* deletion mutants EGP14, EGP17, mut3-98 and control (deposited 81Gb, 82Gb, 77Gb and 79Gb, respectively)	This paper	NCBI Bioproject: PRJNA474609
Bisulfite-treated genome sequence data of *S. latifolia* Y-deletion mutants ESS1, ESS4 and control (deposited 26Gb, 25Gb and 26Gb, respectively)	This paper	NCBI Bioproject: PRJNA474609
Transcriptomic data of 18 *S. latifolia* deletion mutants and controls (Data S1)	This paper	NCBI Bioproject: PRJNA474609
Transcriptomic data of parents and F2	[13]	NCBI Bioproject: PRJNA289919

**EXPERIMENTAL MODELS: ORGANISMS/STRAINS**		

*S. latifolia* deletion mutants	This paper	Listed in Table S1

**SOFTWARE AND ALGORITHMS**		

BWA v.0.7.12-r1039	[44]	http://bio-bwa.sourceforge.net/
Samtools v.1.2.1	[45]	http://www.htslib.org/doc/samtools.html
RSEM v.1.2.31	[46]	https://github.com/deweylab/RSEM
Bowtie2 v.2.1.0	[47]	http://bowtie-bio.sourceforge.net/bowtie2/index.shtml
GenomeAnalysisTK v.3.4-46	[48]	https://software.broadinstitute.org/gatk/
GSNAP v.2018-02-12	[49]	https://github.com/juliangehring/GMAP-GSNAP
Bismark v.0.20.1	[50]	https://www.bioinformatics.babraham.ac.uk/projects/bismark/
DelMapper	[14]	https://github.com/ion-beam-breeding/DelMapper
R v.3.4.4	[51]	https://www.r-project.org/

### Contact for Reagent and Resource Sharing

Further information regarding the manuscript and requests for reagents may be directed to, and will be fulfilled by the Lead Contact, Dmitry A. Filatov (Dmitry.Filatov@plants.ox.ac.uk).

### Experimental Model and Subject Details

In this study we used mutant and wild-type *Silene latifolia* plants all of which belonged to the same highly inbred K-line that was generated by full-sib mating for 14 generations by Kazama and colleagues [52]. The draft genome sequence of that inbred line was published previously [13]. The 101 Y-deletion mutants used for deletion mapping of the Y chromosome and the phenotypes of these mutants are listed in Table S1. All plants used in the study were grown in the glasshouse at ambient temperature with long day (16h) lighting regime.

### *Method* Details

#### Deletion mutants

All the mutants that were used in this study were generated using the methodology described previously [14]. Briefly, pollen grains or dry seeds of the inbred K-line were irradiated with carbon ion beams with a linear energy transfer (LET) of 30 keV μm^-1^ or iron ion beams with a LET of 640 keV μm^-1^ to generate random deletions. The tissue and dose of irradiation used for each mutant are listed in Table S1. Heavy-ion beams have high LET and thus are expected to induce more localized deletions than low-LET radiation, such a X rays or γ rays [53]. The mutants were found by changes in the floral phenotypes observed in the M1 or M2 generations. The deletions were identified and verified by PCR and sequencing. The PCR primers used for this purpose are listed in Table S2.

A subset of the mutants used for Y-deletion mapping for which plants were still alive and actively growing at the time of the study, were used for high throughput sequencing (Table 1). The Y-deletion mutants sequenced in this study can be divided into groups according to the deletion map on the Y chromosome and the phenotypic effect of the deletion (Figure 2A and Table S1). The first group comprised hermaphroditic mutants (m441, EGP4, EGP14, EGP15, EGP16 and EGP17) which lost the gynoecium suppressing function (GSF) gene and a number of adjacent genes. The second group includes asexual mutants (K034, ESS1, ESS4, ESS5, ESS7, ESS8, ESS10 and ESS11) which lost the stamen promoting function (SPF) gene and adjacent genes. One of these asexual mutants, ESS10 also contained a large deletion in the q arm of the Y chromosome, which allowed us to test whether the deletions in the q arm of the Y chromosome trigger IDC. In addition to that, we analyzed a multiple deletion mutant (GPSS1) which lost both GSF and SPF and their adjacent genes and two Y-deletion mutants with normal male phenotype (M51 and mut3-70) that contained deletions in the q arm of the Y chromosome.

#### Deletion mapping of the Y chromosome

Construction of the Y-deletion map for the *S. latifolia* inbred K-line followed the same methodology as described previously [14]. Briefly, 163 STS markers were used to determine the location of Y deletions in the mutants (Tables S1 and S2). The resulting marker presence/absence data was input into DelMapper program [14] with the “Any” option and the option to specify the number of the marker clusters as 14. Under these options, the markers were clustered into 14 clusters and the markers in each cluster were summarized in a virtual marker. DelMapper was used to calculate the most plausible order(s) of the virtual markers. In each cluster, DelMapper calculated the most plausible order(s) of the markers recursively. In the calculations, the ends of the mapping region were assumed to have same deletion status (present or absent) as the adjacent marker.

#### Genomic sequencing

To facilitate the identification and verification of deleted genes we sequenced the genomes of three deletion mutants (EGP14, EGP17 and mut3-98) and of a non-irradiated male plant of the K-line. In addition to that, we used genome sequence data from non-irradiated plants of the inbred K-line that were published previously [13]. DNA for genomic sequencing was extracted from fresh leaves using the DNeasy Plant Mini kit (QIAGEN). For high-throughput sequencing, PCR-free Illumina libraries were prepared at Oxford Wellcome Trust genomic center (WTCHG). These libraries were sequenced on HiSeq2500 instrument at WTCHG. Reads were aligned against the reference [13] with BWA v.0.7.12 (bwa aln, standard parameters except -n 1) [44] and the read alignment files were processed with Samtools v.1.2 [45] to remove duplicate reads (with mapping quality threshold = 20) and obtain the coverage for different regions and genes across the genome (using Samtools idxstats).

#### Transcriptome sequencing

Mutant and control plants used for transcriptome sequencing were grown in the glasshouse with standard long day (16h light) conditions. RNA was extracted from fresh leaves and flower buds with the RNeasy Plant Mini Kit (QIAGEN), including the DNase treatment step recommended by the QIAGEN manual for that kit. Transcriptome sequencing libraries were prepared with Illumina TruSeq mRNA Library Prep Kit at the WTCHG. The sequencing of the libraries was conducted at the WTCHG on Illumina HiSeq4000 instrument with 75 bp paired-ends reads. Up to three technical replicates per plant were extracted at different times and sequenced separately to minimize expression noise (Data S1). Reads were aligned against the reference transcriptome [13] with Bowtie2 v.2.1.0 with standard parameters (-n 2 -l 25 -I 1 -X 1000 -p 1 -a -m 200) and expression values were obtained for each gene using RSEM v.1.2.31 [46] with default parameters to extract the FPKM values.

#### Identification of deleted genes for expression analyses

A previous study [14] identified and verified 71 loci deleted in the Y-deletion mutants; 35 of these genes were used in our expression analyses. The rest of the previously verified Y-linked deletions could not be used due to unavailability of plant material for these mutants. Using genomic sequence data for mutant males (EGP14 and EGP17) and non-irradiated controls we double-checked the Y deletions identified previously [14]. As expected, the Y gametologs had zero coverage in the deletion mutants. The gene expression level was used as another validation of the deleted Y-linked genes, which confirmed zero coverage for previously verified Y-linked genes in the deletion mutants, while the X-copy (in mutants and non mutants) and the Y-copy in non mutants were expressed (Data S1). By extension, we identified new Y deletions if (*i*) the Y gametolog had a coverage of zero and (*ii*) the X gametolog was expressed in at least a subset of mutants, and if (*iii*) both X and Y gametologs were expressed in non mutant individuals.

The pollen grain nuclei are haploid, so a deletion of a Y-linked copy of a sex-lined gene due to pollen irradiation leads to a single maternal X-linked gametolog present in the adult male progeny. Similarly, for autosomal deletions the mutant is heterozygous for the deletion and has a single copy of the genes within the deleted region. This allowed us to use a sequence coverage approach to detect autosomal deletions, where the mutants are expected to have half the coverage of the non mutants, using the RPKM value (Table S3). The RPKM value was obtained for each gene by taking the number of mapped reads divided by the length of the gene and the total million number of mapped reads.

#### Expression analysis of deletion mutants

Expression was quantified with the commonly used measure FPKM (fragments per kilobase per million mapped fragments) value that combines inter- and intra-sample normalization by rescaling read counts per gene to correct for differences in both library size and gene length [15]. This approach was shown to be one of the most accurate ways to normalize RNA-seq data [54]. FPKM values show high correlation between the technical replicates (Pearson correlation, *rho* > 0.82, P value < 2.2x10^−16^), indicating high quality of data acquisition. The average FPKM values across technical replicates for each individual were used for further analyses.

The Y and X gametologs in *S. latifolia* diverged 6 to 11 million years ago, as was recently revealed by direct estimation of the spontaneous mutation rate in this species [9]. As such, the X- and Y-linked gene copies are sufficiently divergent to accurately estimate expression of each copy separately. In particular, as demonstrated in our previous study [13], the Y-linked gametologs show zero expression in females, which indicates no cross-mapping of X reads to Y-linked gametologs. Furthermore, the presence or absence of Y gametologs in the reference sequence during read mapping does not alter the measurements of expression of X-linked gametologs.

The sex-linkage of genes used in the analyses was established in our previous work [10, 13]. We defined *X*_*del*_ and *X*_*nondel*_ as the level of expression of the X gametologs with deleted and non-deleted Y gametologs, respectively, in the deletion mutant, while *X*_*c*_ denotes the expression of the X-linked genes in the controls. We compared the expression level of the X gametolog between mutants (*X*_*del*_ or *X*_*nondel*_) and control plants (*X*_*c*_), calculating the *X*_*del*_*/X*_*c*_ or *X*_*nondel*_*/X*_*c*_ ratios for each gene. Sex-linked genes were divided into different categories depending on divergence between homologous X- and Y-linked gene copies (Figures S1A–S1G), the extent of Y degeneration (Figures S1I–S1O), and the position of Y-linked gene in the map of the Y chromosome (Figures 2 and S2). This enabled us to test whether IDC is more pronounced in any of these categories. The divergence between homologous X- and Y-linked gene copies was calculated at synonymous positions (*dS*) using paml [55]. According to *dS* between X- and Y-linked gametologs, sex-linked genes were split into groups with low (*dS*<0.04), intermediate (0.04 < *dS*<0.08) and relatively high (*dS*>0.08) divergence. The degeneration of the Y-linked gametologs was estimated according to the extent of expression difference between the X and Y gametologs in the controls (*Y*_*c*_*/X*_*c*_); (*i*) genes with a *Y*_*c*_*/X*_*c*_ ratio below 0.3 were considered to have highly degenerate Y gametologs; (*ii*) genes with a *Y*_*c*_*/X*_*c*_ ratio between 0.3 and 0.7 were considered to have partially degenerated Y gametologs; (*iii*) genes with a *Y*_*c*_*/X*_*c*_ ratio above 0.7 were treated as having non-degenerate Y gametologs.

#### Methylation analysis

In order to test whether methylation has a role in upregulation of *X*_*del*_ genes in *S. latifolia*, we analyzed genomic sequences of bisulfite-treated DNA of two mutants ESS1 and ESS4 and the non-irradiated control. DNA was extracted from leaves with the DNA plant easy kit following standard instructions. Bisulfite treatment, library construction and high-throughput sequencing (150b paired end) on Illumina Novaseq S4 platform was done by Beijing genomic institute. The resulting sequence reads have been aligned against the reference transcriptome and reference genome with Bismark [50] using standard parameters (–comprehensive option). The analysis of methylation was conducted in coding sequences and 5′ regions (upstream 5kb) of the *X*_del_, *X*_nondel_ and autosomal genes used in the study. The global methylation state of CpG, CHH and CHG sites have been extracted with Bismark for autosomal and sex-linked genes with an average coverage ≥ 3. As recommended for such analyses [56], methylation was analyzed only at the positions with minimal coverage of 5. We used the methylation percentage ratio between the mutants and the control to detect a variation in methylation state. The significance of difference was tested with Wilcoxon rank sum test.

#### Genomic imprinting analysis

In order to test whether imprinting is involved in *S. latifolia* dosage compensation, we followed the approach of Muyle et al. [43], but used an independent dataset published previously [13]. The dataset comprised transcriptome data for parents and 52 progeny (20 males and 32 females) of a *S. latifolia* genetic cross [13]. High coverage genome sequence data for parents and grand-parents of that genetic cross were also available [9], which helped to establish the genotypes of alleles segregating in the expressed genes. The sequence reads were mapped against the reference transcriptome [13] using GSNAP [49] with the allele tolerance option (-v) that allows a second reference allele for a site. This avoids any reference allele mapping bias using previously identified SNP as a second reference [13]. The resulting bam files were processed with Samtools [45] and Picardtools (http://broadinstitute.github.io/picard) for a new round of SNP calling using HaplotypeCaller from GATK [48] to get the coverage of each allele of previously identified polymorphic positions. The RPKM values were calculated at each polymorphic position and then the average of allelic expression (*E*) was calculated for each gene in each individual with the same formula that was used by Muyle et al. [43]: *E = r/(n^∗^l)*, where *r* is the total number of reads covering one of the two alleles (paternal and maternal) of a gene, *n* is the number of SNP positions in the gene and *l* the library size. The *E* values are calculated separately for the alleles of maternal and paternal origin (*E*_*maternal*_ and *E*_*paternal*_, respectively) for each gene in each individual. Then, we averaged all *E* values of the same origin across all the progeny of the same sex, which yielded four separate *E*-values per gene: *E*_*maternal_in_females*_, *E*_*paternal_in_females*_, *E*_*maternal_in_males*_ and *E*_*paternal_in_males*_. The distributions of *E*_*maternal_in_females*_ / *E*_*paternal_in_females*_ and *E*_*maternal_in_males*_ / *E*_*paternal_in_males*_ ratios were analyzed separately for X-linked and autosomal genes (Figure S4). Furthermore, following Muyle et al. [43], sex-linked genes were divided into three categories (Y/X > 0.75; 0.75 > Y/X > 0.25; Y/X < 0.25) according to the extent Y degeneration and the relative expression of Y versus X gametologs (Y/X). The expression of the X and Y gametologs was measured previously [13].

### Quantification and Statistical Analysis

Throughout the paper the significance of expression difference between the categories was tested using the two-sided Wilcoxon rank sum test. Relative expression (mutants/controls expression ratios) for each category was summarized as probability density histograms (with kernel smoothing function), as well as in the form of notched box-and-whisker plots generated in R. In the box-and-whisker plots, the box spans the first quartile (Q1) to the third quartile (Q3); the line inside the box shows the median; the notches represent the 95% confidence interval for the median; wiskers show the range between Q1 - 1.5x(Q3 - Q1) and Q3 + 1.5x(Q3 - Q1) and should include 99.3% of the data if it were normally distributed. All the boxplots show the outliers represented with small circles outside the range of the wiskers. The number inside the box lists the number of genes analyzed for each category. Differential expression was tested for every gene with t test comparing expression (FPKM) across the technical replicates for the controls and the particular mutant. The p values of the t tests were corrected for multiple testing using R function *p.adjust* with Benjamini and Hochberg false discovery rate (FDR) correction [57]. The principle components analysis of expression was conducted using R function *prcomp* and the PCA plot (Figure 2B) was generated with *ggbiplot* function from R package *ggbiplot* v0.55. Volcano plots of *X*_*del*_, *X*_*nondel*_ and autosomal genes (Figure S2) were generated using the *volcanoPlot* function in R v3.5.1. In these plots the significance of change in expression between the mutants and controls (y axis) was plotted against log2-transformed extent of expression change (x axis) for each gene. The significance of the expression changes between mutants and controls were tested with *t.test* in R.

### Data and Software Availability

All genomic and transcriptomic raw sequence reads we used are available from NCBI under the Bioprojects PRJNA474609 and PRJNA289919.

## Supplemental Information

Document S1. Figures S1–S4

Table S1. Y-Deletion Mutants and Deletion Map of *S. latifolia* Y Chromosome, Related to Figure 2 The map is based on 101 mutants and 163 Y-linked markers. The presence and absence (deletion) of a marker in the particular mutant is indicated with 1 (white background) and 0 (black background), respectively. The green “Yregions” denote the likely location of genes listed in Data S1. Yregions are shown on two rows to make the borders between adjacent regions more visible. Note that the Yregion 9 is in 3 fragments that cannot be distinguished with the set of deletions analyzed. Red and blue translucent boxes indicate the regions in which GSF and SPF are thought to be located, respectively.

Table S2. List of Y-Linked STS Markers and Primers Used in This Study for Y-Deletion Mapping, Related to Figure 2

Table S3. Genomic and Transcriptomic Coverage of the LG4 Linked Genes in Mutant and Controls, Related to Figure 3 Genes deleted in mut3-98 are shown with a tick.

Data S1. Leaf and Bud Gene Expression Data Used in the Study, Related to Table 1 and Figures 1 and 2 Expression values are measured as FPKM (read fragments per kilobase of reference per million read fragments mapped). Genes deleted on the Y chromosome are highlighed in yellow, while their X gametologs (*X*_del_ genes) are highlighted in green. All other (non-highighted) X-linked genes belong to *X*_nondel_ category. Y-linked genes with deletions are sorted according to their position in the Y-deletion map (Table S1) - the location is shown in column “Yregion,” which corresponds to “Yregion” highlighted in green in Table S1.

Document S2. Article plus Supplemental Information
