# Supplementary material for: Immediate Dosage Compensation Is Triggered by the Deletion of Y-Linked Genes in Silene latifolia
Source: Curr Biol. 2019 Jul 8;29(13):2214–2221.e4. doi: 10.1016/j.cub.2019.05.060 (PMC6616318; doi:10.1016/j.cub.2019.05.060)
Supplement: Document S1. Figures S1–S4 [file mmc1.pdf]

Current Biology, Volume *29*

Supplemental Information

Immediate Dosage Compensation Is Triggered by the Deletion of Y-Linked Genes in *Silene latifolia*

Marc Krasovec, Yusuke Kazama, Kotaro Ishii, Tomoko Abe, and Dmitry A. Filatov

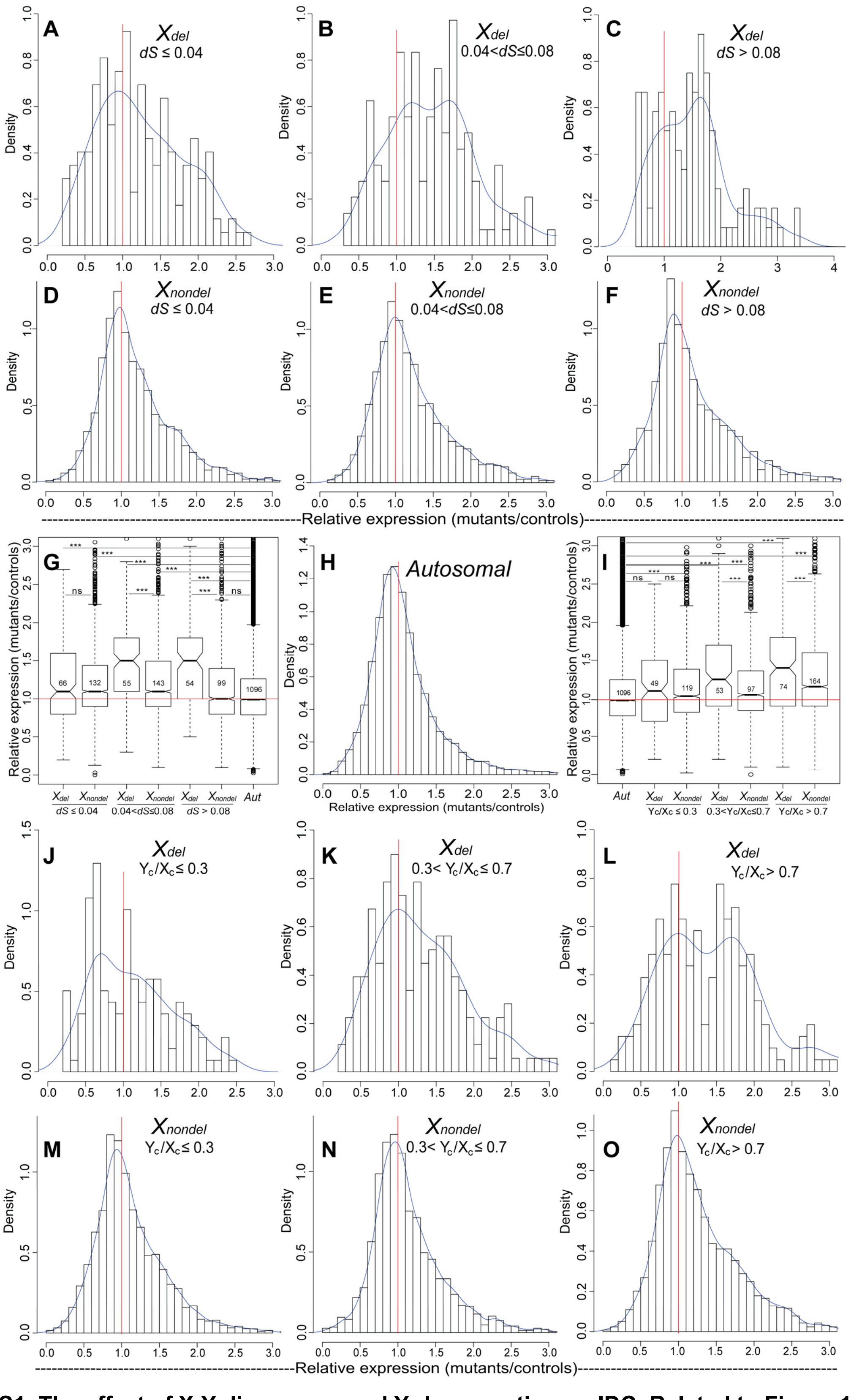

Figure S1. The effect of X:Y divergence and Y-degeneration on IDC. Related to Figure 1. Up-regulation of X_{del} and X_{nondel} gene expression in the deletion mutants compared to non-irradiated controls depending on synonymous divergence (*dS*) between X- and Y-linked gametologs (panels A to G) and the extent of degeneration of their Y-linked gametologs (panels I to O). The extent of Y-degeneration is measured as Y/X expression ratio in controls (Y_c/X_c). Significance of difference between different categories (Wilcoxon rank sum test, *** $P < 0.001$) and numbers of genes analysed are shown in panels G and I.

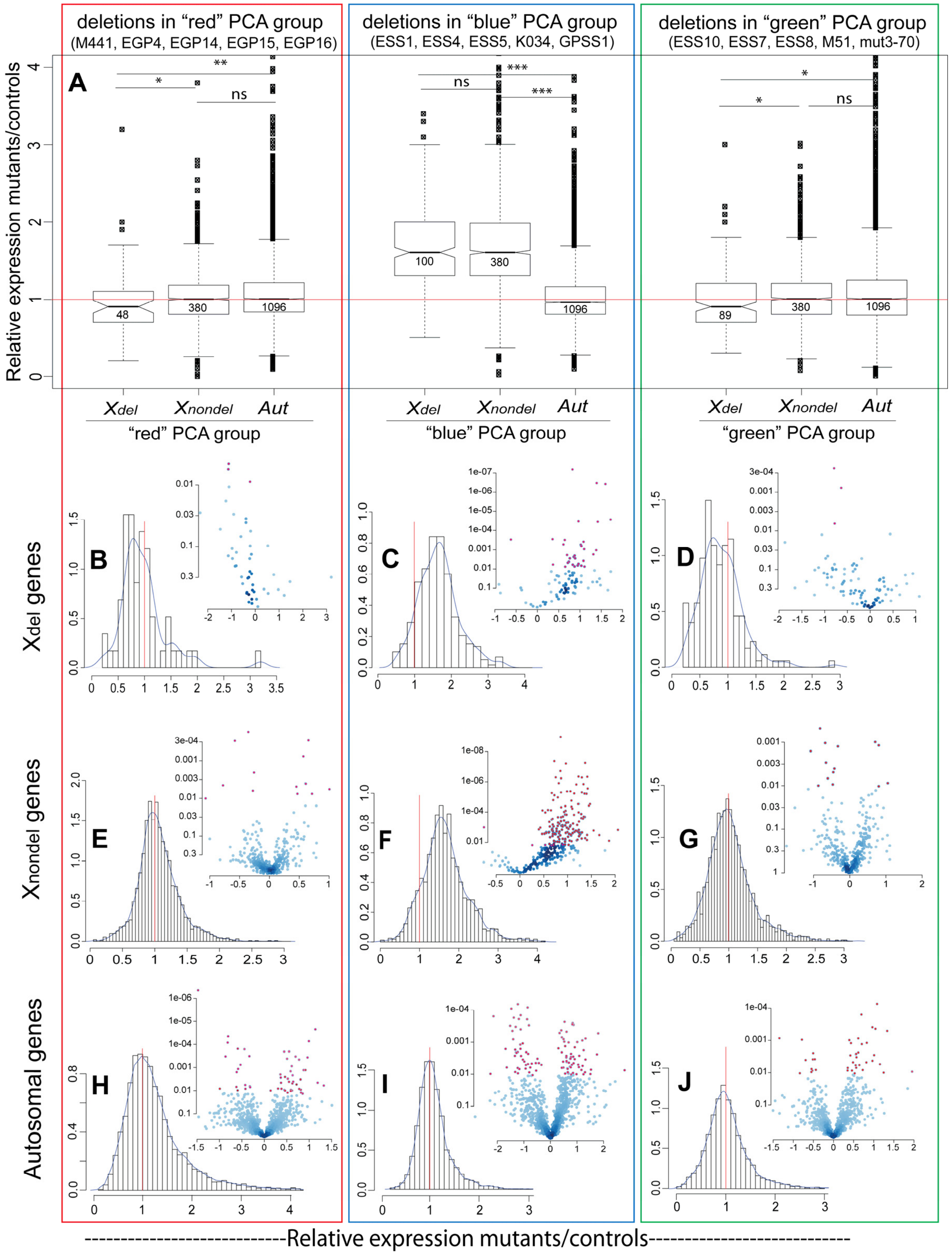

Figure S2. **Up-regulation of X-linked genes is caused by deletions in the mutants of the "blue" PCA cluster**. **Related to Figure 2**.This figure shows the same analyses as in Figure 2C to 2L but with normalisation by non-irradiated mutants. The number of genes in each category is shown within the boxes (panel A). Significance of difference between different gene categories was tested with Wilcoxon rank sum test: * *P*<0.05; ** *P*<0.01; *** *P*<0.001. The insets for each histogram (panels B to J) show volcano plots of significance of expression change (vertical axis) versus log_2-fold change (horizontal axis) in gene expression in mutants relative to controls. The genes showing significant (t-test, *P*<0.01) expression change in mutants compared to controls are highlighted in red.

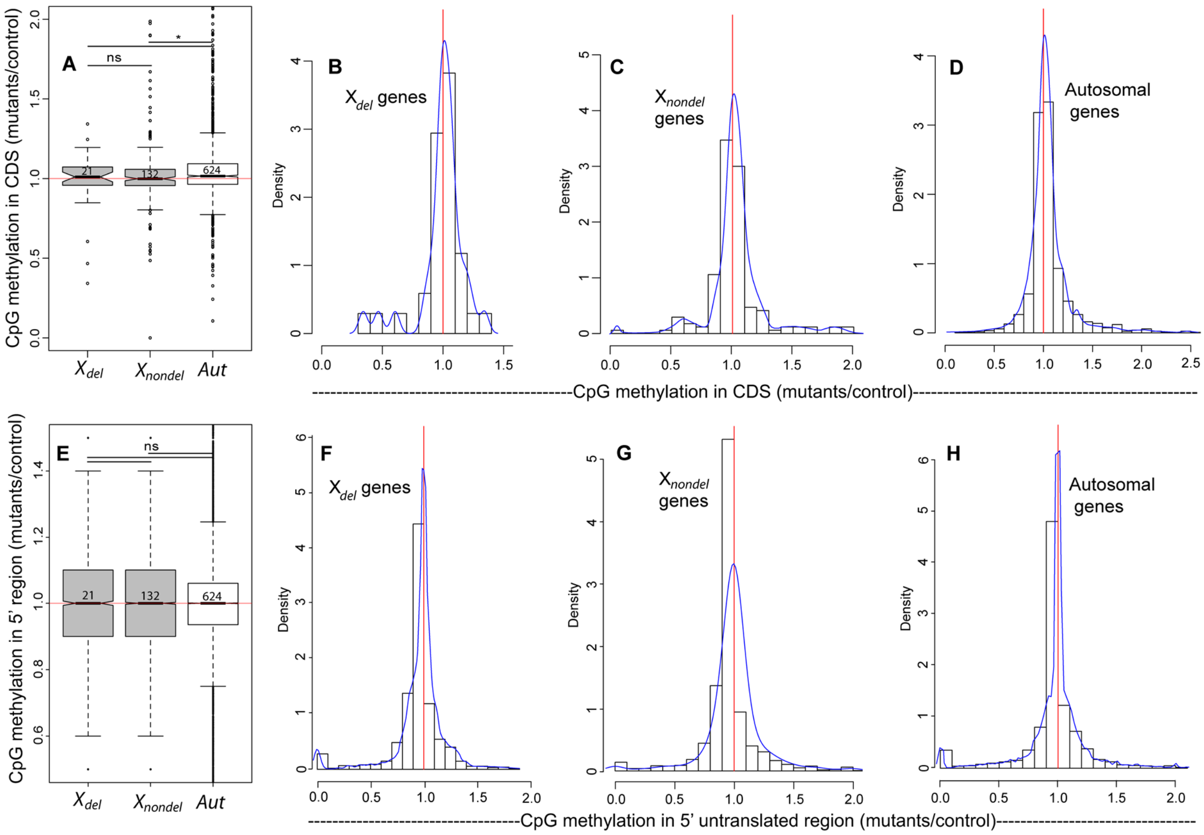

Figure S3. DNA methylation is not involved in IDC. **Related to Figure 2**. DNA methylation in leaves of mutants ESS1 and ESS4 relative to control at CpG sites in coding regions (A to D) and 5' untranslated regions (E to H) of X_{del}, X_{nondel} and autosomal (*Aut*) genes. The red lines in all panels shows the null expectation for no difference between the mutants and control. The blue curve in panels B to D and F to H show the kernel smoothing function. The number of genes analysed in each category is shown within the boxes on the boxplots. Significance of difference between different gene categories was tested with Wilcoxon rank sum test: * P<0.05; ns, non-significant.

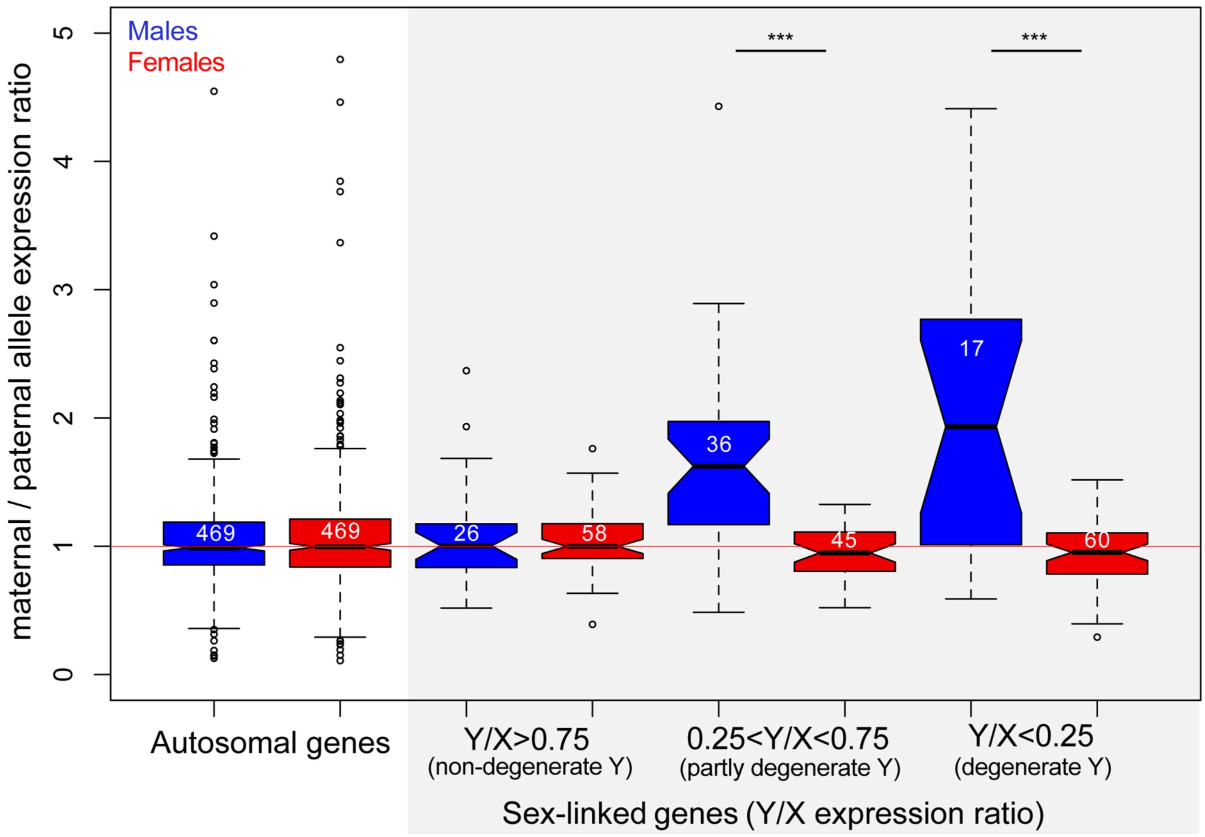

Figure S4. No evidence for maternal imprinting in *S. latifolia*. Related to Figure 2. Transcriptome sequence data from a genetic cross, including parents and 52 progeny (20 males and 32 females) was used to track the inheritance of maternal and paternal alleles and to estimate allele-specific expression as described in the methods. The expression ratios of maternal and paternal alleles in females (red) do not differ significantly from the expectation of equal expression. In males (blue), the deviation from equal expression in two categories of sex-linked genes is caused by Y-degeneration that results in weaker expression of Y-gametologs (paternal) compared to their X-gametologs (maternal) in males. Number of genes analysed in each category is shown inside the boxes on the boxplot. Wilcoxon rank sum test: *** $P < 0.001$.

SUPPLEMENTAL REFERENCE

S1. Kazama, Y., Ishii, K., Aonuma, W., Ikeda, T., Kawamoto, H., Koizumi, A., Filatov, D.A., Chibalina, M., Bergero, R., Charlesworth, D., et al. (2016). A new physical mapping approach refines the sex-determining gene positions on the *Silene latifolia* Y-chromosome. Sci Rep *6*, 18917.
