# Supplementary material for: Immediate Dosage Compensation Is Triggered by the Deletion of Y-Linked Genes in Silene latifolia
Source: Curr Biol. 2019 Jul 8;29(13):2214–2221.e4. doi: 10.1016/j.cub.2019.05.060 (PMC6616318; doi:10.1016/j.cub.2019.05.060)
Supplement: Document S2. Article plus Supplemental Information [file mmc6.pdf]

Report

Current Biology

Immediate Dosage Compensation Is Triggered by the Deletion of Y-Linked Genes in *Silene latifolia*

Highlights

- Dosage compensation on *Silene latifolia* X chromosome can be triggered by Y deletions
- We constructed a new high-density deletion map of *S. latifolia* Y chromosome
- The gene(s) controlling X upregulation map to a region on the p arm of the Y chromosome
- DNA methylation and imprinting are not involved in *S. latifolia* dosage compensation

Authors

Marc Krasovec, Yusuke Kazama, Kotaro Ishii, Tomoko Abe, Dmitry A. Filatov

Correspondence

dmitry.filatov@plants.ox.ac.uk

In Brief

Krasovec et al. use Y deletions to study evolution of dosage compensation on recently evolved sex chromosomes in *Silene latifolia*. They report that experimental deletion of Y-linked genes leads to immediate upregulation of X gametologs, indicating that dosage compensation does not have to evolve gene by gene with progressing Y degeneration.

Krasovec et al., 2019, Current Biology *29*, 2214–2221
July 8, 2019 © 2019 The Authors. Published by Elsevier Ltd.
https://doi.org/10.1016/j.cub.2019.05.060

CellPress

Current Biology
Report

Immediate Dosage Compensation Is Triggered by the Deletion of Y-Linked Genes in *Silene latifolia*

Marc Krasovec,[1] Yusuke Kazama,[2,3] Kotaro Ishii,[2] Tomoko Abe,[2] and Dmitry A. Filatov[1,4,*]
[1]Department of Plant Sciences, University of Oxford, Oxford OX1 3RB, UK
[2]RIKEN Nishina Center for Accelerator-Based Sciences, 2-1 Hirosawa, Wako, Saitama, 351-0198, Japan
[3]Present address: Department of Bioscience and Biotechnology, Fukui Prefectural University 4-1-1 Kenjojima, Matsuoka, Eiheiji-cho, Fukui 910-1195, Japan
[4]Lead Contact
*Correspondence: dmitry.filatov@plants.ox.ac.uk
https://doi.org/10.1016/j.cub.2019.05.060

SUMMARY

The loss of functional genes from non-recombining sex-specific chromosomes [1, 2], such as the Y chromosomes in mammals [3] or W chromosomes in birds [4], should result in an imbalance of gene products for sex-linked genes [5]. Different chromosome-wide systems that rebalance gene expression are known to operate in organisms with relatively old sex chromosomes [6]; e.g., *Drosophila* overexpress X-linked genes in males [7], while mammals shut down one of the X chromosomes in females [8]. It is not known how long it takes for a chromosome-wide dosage compensation system to evolve. To shed light on the early evolution of dosage compensation, we constructed a high-density Y-deletion map and used deletion mutants to manipulate gene dose and analyze gene expression in white campion (*Silene latifolia*), which evolved dioecy and sex chromosomes only 11 million years ago [9]. We demonstrate that immediate dosage compensation can be triggered by deletions in a large portion of the p arm of the Y chromosome. Our results indicate that dosage compensation in *S. latifolia* does not have to evolve gene by gene because a system to upregulate gene expression is already operating on part of the X chromosome, which likely represents an intermediate step in the evolution of a chromosome-wide dosage compensation system in this species.

RESULTS AND DISCUSSION

Very little is known about dosage compensation (DC) in plants [10–13]. The *de novo* evolution of sex chromosomes in *Silene latifolia* (*Caryophyllaceae*) only 11 million years ago [9] offers a rare opportunity to analyze the early stages of sex chromosome evolution, with actively progressing Y degeneration and evolving DC "caught in action." The previous studies on *S. latifolia* [12, 13] reported signs of a nascent DC system upregulating X-linked genes with degenerate or partially degenerate Y-linked gametologs—the homologous genes shared between the X and the non-recombining Y chromosomes. As *S. latifolia* is not easily amenable to transgenic analyses, the molecular bases of this DC remain unexplored. In particular, it remains unclear whether DC has to evolve gene by gene—independently at each sex-linked gene that lost its Y-linked gametolog or if some form of chromosome- or region-wide DC is already present on *S. latifolia* sex chromosomes. Here, we make use of a set of Y-deletion mutants generated in an *S. latifolia* inbred strain [14] to test whether experimental deletion of the Y-linked genes leads to altered expression of their respective X gametologs. If DC in *S. latifolia* is required to evolve gene by gene, then Y-linked gene deletion should not immediately lead to altered expression of the corresponding X gametolog, as there would not have been time for it to evolve. On the other hand, if immediate dosage compensation (IDC) is observable for X gametologs of experimentally deleted Y-linked genes, it would imply that some form of DC is already operating.

Y Deletion Mutants and the Deletion Map of *S. latifolia* Y Chromosome

In this study, we used 101 *S. latifolia* Y-deletion mutants (Table S1), 67 of which have not been described previously. All these mutants were generated by heavy ion irradiation of pollen grains or dry seeds [14]. As the original aim of generating these mutants was to precisely locate the sex-determining gynoecium-supressing function (GSF) and stamen-promoting function (SPF) genes on the Y chromosome, most of these mutants were initially identified by morphological changes in male or female organs in the flowers [14]. As Y-deletion mutants contain Y chromosomes, they are genetically males, but deletions of sex-determining SPF and/or GSF genes altered the presence of stamens and pistils in the flower (Table S1). The presence of Y deletions in each mutant was analyzed by PCR-based genotyping of 163 STS markers (Table S2), with 0 to 81 markers found to be deleted per mutant. The presence of deletions in other chromosomes was analyzed in EGP14, EGP17, and mut3-98 mutants relative to control, for which approximately 80 Gb of sequence data with 150 bp paired-end reads were generated per individual. No deletions outside the Y chromosome were found except one autosomal deletion in linkage group 4 of mutant mut3-98 (see below).

 © 2019 The Authors. Published by Elsevier Ltd.
This is an open access article under the CC BY license (http://creativecommons.org/licenses/by/4.0/).

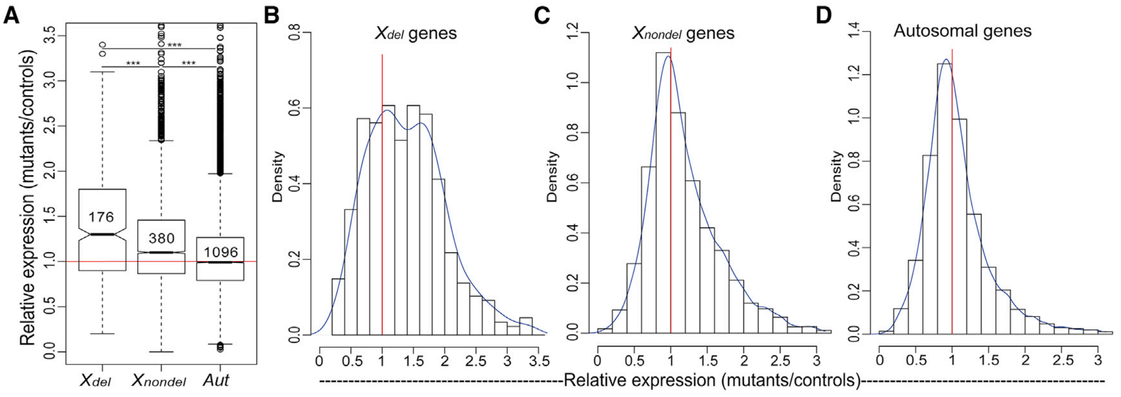

Figure 1. Relative Expression of X-Linked and Autosomal Genes in Leaves of Mutants Compared to Controls

The X-linked genes are split into two categories: the genes for which Y-linked gametologs were deleted (X_{del}) and not deleted (X_{nondel}) in the deletion mutants.

(A) Significant upregulation of X-linked genes in the mutants compared to controls is evident in both categories of X-linked genes, though it is much stronger in the X_{del} compared to X_{nondel} genes. The numbers of genes analyzed in each category are shown within the boxplot. The difference between gene categories was tested for significance with the Wilcoxon rank sum test, ***$p < 0.001$. In the box-and-whisker plot (A), the box spans the first quartile (Q1) to the third quartile (Q3); the line inside the box shows the median; the notches represent the 95% confidence interval for the median; and whiskers show the range between Q1 − 1.5 × (Q3 − Q1) and Q3 + 1.5 × (Q3 − Q1).

(B–D) Distributions of expression change in the mutants compared to controls for X_{del} genes (B), X_{nondel} genes (C), and autosomal genes (D). The curves in (B) to (D) show the kernel-smoothed density function. The red lines at 1 in all panels show the null expectation for no difference in expression between deletion mutants and controls.

Data used for this figure are listed in Data S1. See also Figure S1.

Following the previously described approach [14], we constructed a new high-density deletion map for the *S. latifolia* Y chromosome (Table S1), which is a significant improvement over the previous iteration of a Y-deletion map that was based on only 41 mutants and 71 Y-linked markers [14]. The new map allowed us to locate the Y deletions affecting expression of X-linked genes (see below) as well as narrow down the regions in which the sex-determining genes are located (Table S1). Due to pre-selection of the mutants by morphological changes in male of female organs in the flowers [14], most Y deletions clustered around the two sex-determining genes in the p arm, although a few deletions were also found in the q arm of the Y chromosome (Table S1).

Seventeen of the Y-deletion mutants and three controls plants that were still actively growing at the time of the study were used for high-throughput transcriptome sequencing (Data S1). Using sequence coverage in the sequenced mutant males and non-irradiated controls, we reconfirmed the Y deletions identified previously [14]. As expected, the deleted Y gametologs had zero sequence coverage in the deletion mutants, and active expression was confirmed in the control plants (Data S1). Using this approach, combined with PCR-based verification, we identified 177 Y-linked genes deleted in the irradiated mutants, including 35 such genes identified previously [14]; 127 of these genes were independently deleted in at least two Y-deletion mutants. All of these Y-linked genes have actively expressed X-linked gametologs whose expression was analyzed in controls and deletion mutants (Data S1).

Expression of X-Linked Genes in Y-Deletion Mutants

To study gene expression changes caused by deletion of Y-linked genes, we analyzed transcriptome sequence data for leaves and flower buds of the deletion mutants and controls with up to three technical replicates per sample (Data S1). Gene expression, measured as FPKM (fragments per kilobase per million [15]) values, showed a high correlation between technical replicates (Pearson's $\rho \geq 0.82$; $p < 2.2 \times 10^{-16}$) and between mutants and controls (Pearson's $\rho \geq 0.86$; $p < 2.2 \times 10^{-16}$). The latter indicates that the overall expression is very similar between the mutants and controls, so the FPKM normalization across samples is adequate, and no external spike in normalization [16] was necessary, as has been done in some studies of chromosomal aneuploidy [17].

In order to compare the effect of the deletion of a Y gene on the expression of the homologous X-linked genes with the effect of the same deletion on the genes for which both copies are present, we split the sex-linked genes into "del" and "nondel" categories (Figure 1). Only the X-linked genes with Y gametolog deleted in the particular mutants were included in the X_{del} category. Similarly, the X_{nondel} category included X-linked genes with the Y gametolog non-deleted in the particular mutants. We first focused on the general pattern of gene expression following the deletion, regardless of the degeneration state of the Y gametolog and its location on the chromosome. The X_{del} genes significantly increased their expression compared to expression of the same genes in the control plants without Y deletions (Figure 1A), and this increase is significantly stronger compared to X_{nondel} and *Aut* genes (Figure 1A; Wilcoxon rank sum tests, $p < 0.001$ for all comparisons). This result indicates the presence of some form of IDC in *S. latifolia*. Interestingly, the distribution of X_{del} expression change in the mutants compared to controls is bimodal, with peaks at around 1 and 1.8 (Figure 1B), which contrasts with unimodal distributions for X_{nondel} and *Aut* genes (Figures 1C and 1D, respectively). This indicates that a fraction of X_{del} genes

nearly double expression in the mutants, while other X_{del} genes do not change expression. A weaker but still significant increase in expression was also observed for the X_{nondel} genes (Figure 1A), indicating that the effect of Y deletions may extend beyond the X_{del} genes. This contrasted with little change in the deletion mutants compared to control plants for autosomal genes (Figure 1A).

The genes of *S. latifolia* sex chromosomes fall into at least two evolutionary strata—the regions of similar divergence between the X and Y gametologs created by stepwise expansion of the non-recombining region on the Y chromosome [13, 18]. The silent divergence between the X and Y gametologs and the extent of Y degeneration is somewhat higher in the older (~11 million years [my] [9]) compared to younger (~6 my [9]) evolutionary stratum [9, 13, 19]. Grouping genes by the extent of synonymous divergence (*dS*) between the X and Y gametologs (Figures S1A–S1G) or by the extent of degeneracy of Y-linked genes (measured by Y_c/X_c expression ratio in controls) deleted in the mutants (Figures S1I–S1O) revealed the presence of IDC for all categories of X_{del} genes, though upregulation was stronger in non-degenerate Y gametologs ($Y_c/X_c > 0.7$, Figure S1) and in genes with $dS > 0.04$ (Figure S1). Weaker but still significant upregulation in mutants compared to that in controls was also observed in most categories of X_{nondel} genes (Figure S1).

IDC Is Caused by Y Deletions Located in a Region between the SPF and GSF Genes

To test whether the location of deletions on the Y chromosome affects IDC, we used our new high-density deletion map of the Y chromosome (Table S1). A subset of the Y-deletion map, including the mutants for which expression was analyzed in the current study, is shown in Figure 2A. Principal components analysis (PCA) of gene expression in the mutants relative to that in controls revealed that mutants form three distinct clusters (hereby referred to as red, blue, and green; Figure 2B) according to the location of Y deletions in the Y chromosome. The analysis of relative expression for each mutant separately (Table 1) revealed the presence of IDC only in the five mutants of the blue PCA cluster (GPSS1, ESS1, ESS4, ESS5, and K034) that have deletions along a large part of the p arm of the Y chromosome. In contrast, deletions elsewhere on the Y chromosome do not appear to trigger IDC (Table 1 and Figure 2). This result is consistent across the tissues analyzed (Table 1).

The five mutants of the blue cluster show IDC in both the X_{del} and X_{nondel} genes, while Y-linked or autosomal genes show no upregulation compared to controls (Figure 2 and Table 1). This indicates that the mechanism responsible for upregulated X expression acts similarly on X_{del} and X_{nondel} genes, but its action is limited to X-linked genes. Using the Y-deletion map, it is possible to locate the factor(s) responsible for triggering X upregulation. The Y region deleted in all five blue cluster mutants, but not in any other mutants, is located proximally to GSF and distally to SPF regions and includes only 13 Y markers, bordered by Ycontig1367 and BenM_cnt21228 (the "IDC-factor" region on Figure 2A). However, it is likely an oversimplification that only deletions in the IDC-factor region trigger IDC, because significant up- or downregulation is also observed for a number of X_{del}, X_{nondel}, and *Aut* genes in the mutants of all three PCA clusters, as revealed by the volcano plots in Figure S2.

Is IDC Specific to the X Chromosome?

Although dosage compensation systems are usually discussed in the context of sex chromosomes, variation in gene dosage can also be found elsewhere in the genome [20]. For example, partial dosage compensation for gene duplications or deletions has been found on *Drosophila* autosomes [21] and in species that have no sex chromosomes [17, 22, 23]. The role of such compensatory upregulation is not well understood and may partly account for dosage compensation in *S. latifolia* and in other plant and animal species.

In order to test whether the IDC can also be triggered on *S. latifolia* autosomes, we analyzed gene expression in an autosomal deletion mutant mut3-98 that was generated via heavy ion irradiation of pollen of an inbred *S. latifolia* strain in the same way as in the Y-deletion mutants described above. To identify the genes deleted in mut3-98, we sequenced its genome with the Illumina HiSeq2500 platform and compared sequence coverage in the mutant and non-irradiated control (Figure 3A and Table S3). This analysis identified 49 autosomal genes that are hemizygous in the mutant as a result of a heterozygous deletion on linkage group 4 (LG4) of the previously published genetic map [13]. Note that only genes present in the map were used in this analysis, as the location of the other genes is unknown. The deleted genes are located in a single contiguous region that is 47.17 cM long and therefore comprises a large portion of LG4 (Figure 3A). The genetic map was used to establish the location of the deleted genes because the genome assembly for *S. latifolia* remains highly fragmented [9, 13]. To test for dosage compensation in the 49 deleted autosomal genes, expression of each hemizygous gene within the heterozygous autosomal deletion (A_{del}) was compared with the expression of the same gene in the controls that were homozygous for no deletion (A_cA_c). The null expectation for genes with no dosage compensation is $A_{del} \sim A_cA_c/2$, while $A_{del} \sim A_cA_c$ is expected for genes with dosage compensation. The observed A_{del} expression did not differ significantly from the expectation for no dosage compensation (Wilcoxon test; W = 692, p = 0.44, ratio A_{del}/A_cA_c compared to 0.5; Figures 3B–3D), indicating an absence of dosage compensation in this part of the genome and possibly elsewhere outside the sex chromosomes in *S. latifolia*. Furthermore, the distribution of A_{del} relative expression did not significantly differ for the autosomal genes inside and outside the deleted region in mut3-98 (Wilcoxon test; W = 2,575, p = 0.17; Figure 3B). Thus, unlike the deletions on the Y chromosome, the deletion of autosomal genes does not trigger IDC in this region, indicating that the mechanism(s) responsible for immediate upregulation of gene expression may be active only on the sex chromosomes of *S. latifolia*. However, it is worth noting that the analysis of a single autosomal deletion cannot rule out the presence of IDC on other autosomes. Indeed, the inverse effect of chromosome dosage on gene expression was described for aneuploids in several other animal and plant species [17, 21–23], which suggests the generality of this phenomenon. Unfortunately, no other autosomal deletions are available for *S. latifolia* to test the presence of IDC in other parts of the genome.

What Are the Molecular Bases of Dosage Compensation in *S. latifolia*?

We demonstrated that expression of *S. latifolia* X-linked genes can be upregulated by experimental deletion of Y-linked

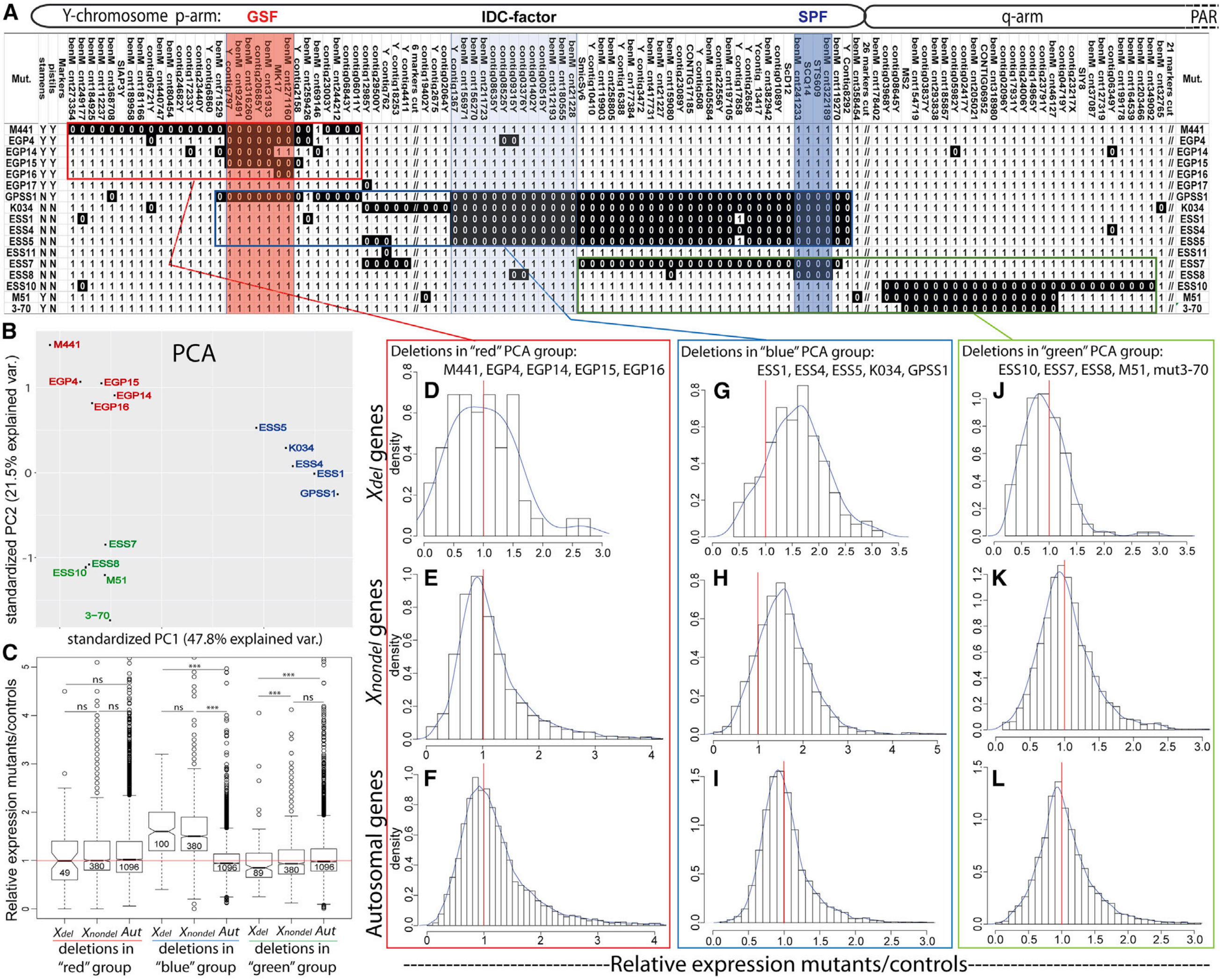

Figure 2. Upregulation of X-Linked Genes Is Caused by Deletions in the IDC-Factor Region of the P Arm of the Y Chromosome

(A) Location of Y deletions in the mutants with sequenced transcriptomes (the full map is shown in Table S1). The presence and absence (deletion) of a marker in the particular mutant is indicated with 1 (white background) and 0 (black background), respectively. Note that 53 uninformative Y markers were excluded (marked with "//") to make this figure narrower. Primers used for genotyping Y markers are listed in Table S2.

(B) Clustering of mutants by relative expression in principle components analysis (PCA).

(C–L) Boxplot comparing relative expression for the same categories of genes as shown in (D) to (L). The significance for these comparisons was tested with Wilcoxon rank sum test, ***$p < 0.001$. The numbers of genes analyzed in each category are shown within the boxes on the boxplot. The box in this box-and-whisker plot (C) spans the first quartile (Q1) to the third quartile (Q3); the line inside the box shows the median; the notches represent the 95% confidence interval for the median; and whiskers show the range between Q1 − 1.5 × (Q3 − Q1) and Q3 + 1.5 × (Q3 − Q1). The red lines in (C) to (L) show the null expectation for no difference in expression between deletion mutants and controls. The blue curves in (D) to (L) show the kernel-smoothed density function for the distributions of relative expression in each category. To control for expression differences that could be caused by deletion of GSF or SPF loci and associated morphology changes, the hermaphroditic EGP mutants were normalized by expression in hermaphroditic mutant EGP17 that contained no big deletions and asexual ESS and K034 mutants were normalized by expression in asexual mutant ESS11 that also lacked big deletions, while q-arm mutants M51 and 3-70 that had a normal male phenotype were normalized by non-irradiated controls. The same analysis with all mutants normalized by non-irradiated controls is shown in Figure S2. Expression data used for these analyses are listed in Data S1. See also Figures S3 and S4 for related analyses testing whether upregulation of X-linked genes is caused by methylation changes or imprinting.

gametologs. Transcriptional buffering [21]—passive attenuation of gene dosage changes due to non-linear properties of the transcription system [24]—is probably the simplest explanation for such IDC. However, the transcriptional buffering hypothesis does not explain why IDC is triggered only by the deletions in the Y chromosome IDC-factor region on Figure 2A. A related mechanism—simultaneous change in gene dose of a negative regulator(s) and the target genes [16, 25–28]—was proposed to explain dosage compensation in aneuploids in many organisms [29–32], including in the species without sex chromosomes, such as maize [22] and *Arabidopsis* [17]. If this mechanism is responsible for IDC in *S. latifolia*, the deletions in the IDC-factor region cause IDC because they reduce the dosage of some gene(s) that negatively regulate(s) expression

CellPress

Table 1. Gene Expression in the Mutants Relative to Non-Irradiated Controls

Mutants:	ESS1	ESS4	ESS5	K034	GPSS1	M441	EGP4	EGP14	EGP15	EGP16	EGP17	ESS11	ESS7	ESS8	ESS10	M51	mut3-70
Leaf Expression in the Mutants Relative to Controls (median ± SD)																	
Xdel	**1.6 ± 0.64**	**1.6 ± 0.51**	**1.5 ± 0.44**	**1.7 ± 0.42**	**1.8 ± 0.78**	0.7 ± 0.36	0.9 ± 0.42	0.7 ± 0.02	0.9 ± 1.16	N/A	N/A	N/A	0.9 ± 0.31	N/A	0.8 ± 0.32	0.9 ± 0.36	0.8 ± 0.51
Xnondel	**1.7 ± 0.66**	**1.6 ± 0.55**	**1.5 ± 0.61**	**1.6 ± 0.45**	**1.7 ± 0.84**	0.9 ± 0.39	0.9 ± 0.31	1.0 ± 0.32	1.0 ± 0.28	1.0 ± 0.28	1.0 ± 0.45	1.1 ± 0.41	1.0 ± 0.47	0.9 ± 0.35	1.0 ± 0.29	1.0 ± 0.47	1.0 ± 0.54
Ynondel	1.0 ± 0.64	1.0 ± 0.48	0.9 ± 0.47	1.0 ± 0.34	1.0 ± 0.53	0.9 ± 0.45	1.0 ± 0.34	1.0 ± 0.36	1.0 ± 0.30	1.0 ± 0.33	0.9 ± 0.44	1.0 ± 0.42	1.0 ± 0.43	0.9 ± 0.32	1.0 ± 0.40	0.9 ± 0.61	0.9 ± 0.62
Autosomal	1.0 ± 0.43	1.0 ± 0.33	0.9 ± 0.39	0.9 ± 0.28	1.0 ± 0.41	1.0 ± 0.41	0.9 ± 0.37	1.1 ± 0.41	1.0 ± 0.39	1.0 ± 0.38	1.0 ± 0.57	1.0 ± 0.40	1.0 ± 0.39	0.9 ± 0.27	1.0 ± 0.34	1.0 ± 0.81	1.0 ± 0.80
Number of Genes Analyzed for Leaf Expression: Autosomal = 1,096 Genes Analyzed in Each Mutant																	
“del”	58	47	50	71	65	43	9	2	4	0	0	0	29	0	60	46	39
“nondel”	322	333	330	309	315	335	371	378	376	380	380	380	351	380	320	334	341
Bud Expression in the Mutants Relative to Controls (median ± SD)																	
Xdel	N/A	**1.6 ± 0.56**	**1.6 ± 0.56**	N/A	N/A	0.7 ± 1.39	0.9 ± 0.50	0.6 ± 0.18	1.0 ± 0.63	N/A	N/A	N/A	0.9 ± 0.34	N/A	N/A	N/A	N/A
Xnondel	N/A	**1.6 ± 0.55**	**1.6 ± 0.54**	N/A	N/A	0.9 ± 0.87	1.0 ± 0.31	0.9 ± 0.38	1.0 ± 0.26	1.0 ± 0.24	N/A	N/A	1.0 ± 0.38	N/A	N/A	N/A	N/A
Ynondel	N/A	1.0 ± 0.56	1.0 ± 0.60	N/A	N/A	0.9 ± 0.77	1.0 ± 0.57	0.9 ± 0.55	1.0 ± 0.54	1.0 ± 0.41	N/A	N/A	1.0 ± 0.43	N/A	N/A	N/A	N/A
Autosomal	N/A	0.9 ± 0.51	0.9 ± 0.43	N/A	N/A	0.9 ± 1.89	1.0 ± 0.68	1.0 ± 0.62	1.0 ± 0.39	1.0 ± 0.37	N/A	N/A	1.0 ± 0.42	N/A	N/A	N/A	N/A
Number of Genes Analyzed for Bud Expression: Autosomal = 1,096 Genes Analyzed in Each Mutant																	
“del”	N/A	48	51	N/A	N/A	43	9	2	4	0	N/A	N/A	30	N/A	N/A	N/A	N/A
“nondel”	N/A	333	330	N/A	N/A	338	372	379	377	381	N/A	N/A	351	N/A	N/A	N/A	N/A

Gene categories significantly upregulated in the mutants are shown in bold ($p < 0.01$; Wilcoxon test). Data used for this table are listed in Data S1.

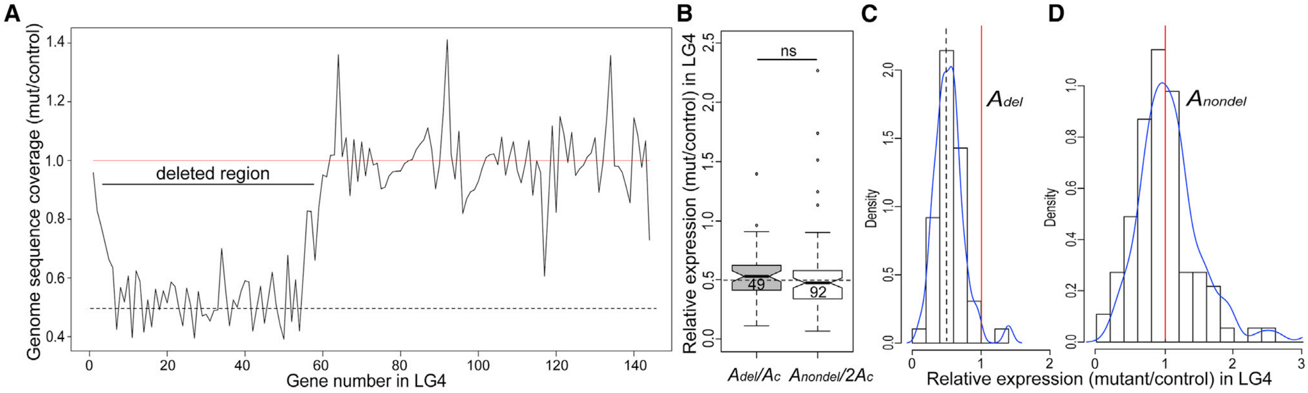

Figure 3. No Evidence for Dosage Compensation in Genes Deleted from Autosomal Linkage Group 4 (LG4) in Leaf Transcriptomes

(A) Graph showing genome sequencing coverage for LG4. The genes are ordered according to their position on LG4.

(B–D) The autosomal genes within the heterozygous deletion (A_{del}) are expressed at ~50% of their total expression in controls (A_c) that are homozygous for no deletion, indicating no dosage compensation in this region. A_{nondel} denotes autosomal genes on LG4 outside the deletion in the mutant mut3-98. A_{nondel} expression for each gene was halved, and A_{del} and A_{nondel} values were normalized by expression of the same genes in the controls. The numbers of genes analyzed in each category are shown inside the boxes. The box in this box-and-whisker plot (B) spans the first quartile (Q1) to the third quartile (Q3); the line inside the box shows the median; the notches represent the 95% confidence interval for the median; and whiskers show the range between Q1 – 1.5 × (Q3 – Q1) and Q3 + 1.5 – (Q3 – Q1). (C) and (D) show the probability density histograms (with kernel-smoothing function) for A_{del} and A_{nondel} genes, respectively. (B), (C), and (D) are based on the same gene expression data in the mutant mut3-98 relative to control. The dashed line at 0.5 in (A), (B), and (C) show the expectation for deleted (hemizygous) region, while the red line at 1 in (A), (C), and (D) shows the expectation for non-deleted region.

Data are listed in Table S3.

of many X-linked genes, while deletions elsewhere on the Y chromosome do not trigger IDC because they do not affect such negative regulator(s). However, this mechanism does not explain why deletions on the Y chromosome primarily affect X-linked genes, while their *trans* effect on autosomal expression is weak. This suggests that IDC may act via some cis-mechanism on the sex chromosomes, such as alteration of chromatin state to enhance the X chromosome chromatin accessibility [33], not dissimilar to that reported for animal dosage compensation systems [34–37]. Alternatively, the IDC could work via re-location of the X chromosome inside the nucleus [35–37], resulting in alteration of the 3D chromatin architecture, as recently reported for plant genomes [38, 39]. Furthermore, these *cis*-acting mechanisms may work in combination with a *trans*-acting negative regulator of expression, as proposed for *Drosophila* [25], that results in two-fold X upregulation and a weaker autosomal effect. Indeed, the volcano plots reveal a small fraction of autosomal genes that are significantly up- or downregulated (Figure S2).

DNA methylation is known to be involved in gene regulation [40] on sex chromosomes and autosomes of many species [41] and was suggested to play a role in sex chromosome evolution in *S. latifolia* [42]. If DNA methylation plays a role in IDC, we expect methylation to differ between the mutants and controls, particularly so at the X_{del} and X_{nondel} genes for deletions in the blue PCA cluster mutants. In order to test this, we compared the extent of DNA methylation between ESS1 and ESS4 deletion mutants and control plant using genomic sequencing of bisulphite-treated DNA (25 to 26 Gb of sequence data per plant). Methylation was detected mainly at CpG sites, while only ~2% of CHH and CHG sites were methylated in the samples analyzed. The analysis of coding regions and the adjacent 5′ untranslated regions revealed no difference in methylation between the mutants and control (Figure S3). Thus, DNA methylation is unlikely to be involved in *S. latifolia* IDC. This result is consistent with the analysis of methylation changes in *A. thaliana* aneuploids, which concluded that "genetic imbalance is generally mechanistically unrelated to DNA methylation" [17].

Based on the analysis of allele-specific expression in females, a recent study reported that dosage compensation in *S. latifolia* acts via maternal imprinting, with the X chromosome inherited from the mother upregulated in both male and female progeny [43]. Such X-chromosome-wide upregulation would result in overcompensation for all X-linked genes in females and for X-linked genes with functional Y gametologs in males. In males, this overcompensation may be ameliorated by Y-linked factor(s) reducing X expression. Deleting these X-suppressing Y-linked factor(s) in Y-deletion mutants may be the cause of IDC detected in our study. Thus, if the *S. latifolia* X chromosome is indeed upregulated, as reported by Muyle et al. [43], the IDC may be a secondary adaptation aimed at preventing overcompensation of sex-linked genes in males. However, re-analyzing published transcriptome sequence data [13], which was obtained independently from the data of [43], we were unable to confirm the conclusion of Muyle et al. [43] that dosage compensation in *S. latifolia* acts via maternal imprinting. Our dataset comprised transcriptome data for parents and 52 progeny (20 males and 32 females) of an *S. latifolia* genetic cross [13]. In our re-analysis of allele-specific expression in an *S. latifolia* genetic cross, the maternal and paternal alleles of X-linked genes in females were equally expressed (Figure S4), which contradicts the results of Muyle et al. [43]. Thus, the role of maternal imprinting in *S. latifolia* dosage compensation has to be taken with caution and requires independent verification. Clearly, the molecular bases of dosage compensation in *S. latifolia* require further analysis.

Conclusions

Overall, our results indicate the presence of a pre-existing dosage compensation system in some regions of *S. latifolia* sex chromosomes. This system can upregulate X-linked genes immediately after loss of their Y-linked gametologs. Thus, many X-linked genes do not have to evolve dosage compensation in a gene-by-gene fashion as Y-linked gametologs degenerate. The immediate dosage compensation of X-linked genes appears to be triggered by deletions in a part of the p arm of the Y chromosome—the IDC-factor region—that likely contains gene(s) controlling expression of the X chromosome. The finding of IDC on the *S. latifolia* sex chromosomes is surprising, given the recent origin of dioecy and sex chromosomes in this species. IDC described in this study may represent an intermediate step in the evolution of a chromosome-wide dosage compensation system. A scenario of how pre-existing IDC may have played central role in evolution of dosage compensation in *Drosophila* was described by Birchler [25]. Similarly, IDC may be the basis of evolving dosage compensation in *S. latifolia*, although the molecular mechanisms of this process remain to be studied.

STAR★METHODS

Detailed methods are provided in the online version of this paper and include the following:

- KEY RESOURCES TABLE
- CONTACT FOR REAGENT AND RESOURCE SHARING
- EXPERIMENTAL MODEL AND SUBJECT DETAILS
- *METHOD* DETAILS
  - Deletion mutants
  - Deletion mapping of the Y chromosome
  - Genomic sequencing
  - Transcriptome sequencing
  - Identification of deleted genes for expression analyses
  - Expression analysis of deletion mutants
  - Methylation analysis
  - Genomic imprinting analysis
- QUANTIFICATION AND STATISTICAL ANALYSIS
- DATA AND SOFTWARE AVAILABILITY

SUPPLEMENTAL INFORMATION

Supplemental Information can be found online at https://doi.org/10.1016/j.cub.2019.05.060.

ACKNOWLEDGMENTS

This work was supported by a grant from the BBSRC (grant BB/P009808/1 to D.A.F.) and JSPS KAKENHI (grant number 15KK0264 to Y.K.). We are grateful to Michael Chester for helpful comments and proofreading of the manuscript. We thank two anonymous reviewers, whose comments helped to improve the manuscript, and the staff at the Wellcome Trust Centre (Oxford) for carrying out the high-throughput sequencing and initial data processing.

AUTHOR CONTRIBUTIONS

D.A.F. conceived the project, designed the work, generated genome and transcriptome sequence data, and drafted the text of the manuscript. M.K. analyzed the data and contributed to writing the manuscript. Y.K. and T.A. generated the deletion mutants and verified the deleted genes with PCR. Y.K. and K.I. constructed the Y-deletion map. All the authors contributed to writing and editing the manuscript.

DECLARATION OF INTERESTS

The authors declare no competing interests.

Received: December 10, 2018
Revised: February 27, 2019
Accepted: May 24, 2019
Published: June 20, 2019

REFERENCES

1. Charlesworth, B., and Charlesworth, D. (2000). The degeneration of Y chromosomes. Philos. Trans. R. Soc. Lond. B Biol. Sci. *355*, 1563–1572.
2. Bachtrog, D. (2013). Y-chromosome evolution: emerging insights into processes of Y-chromosome degeneration. Nat. Rev. Genet. *14*, 113–124.
3. Bellott, D.W., Hughes, J.F., Skaletsky, H., Brown, L.G., Pyntikova, T., Cho, T.J., Koutseva, N., Zaghlul, S., Graves, T., Rock, S., et al. (2014). Mammalian Y chromosomes retain widely expressed dosage-sensitive regulators. Nature *508*, 494–499.
4. Zhou, Q., Zhang, J., Bachtrog, D., An, N., Huang, Q., Jarvis, E.D., Gilbert, M.T.P., and Zhang, G. (2014). Complex evolutionary trajectories of sex chromosomes across bird taxa. Science *346*, 1246338.
5. Mank, J.E. (2013). Sex chromosome dosage compensation: definitely not for everyone. Trends Genet. *29*, 677–683.
6. Ercan, S. (2015). Mechanisms of x chromosome dosage compensation. J Genomics *3*, 1–19.
7. Conrad, T., and Akhtar, A. (2012). Dosage compensation in *Drosophila melanogaster*: epigenetic fine-tuning of chromosome-wide transcription. Nat. Rev. Genet. *13*, 123–134.
8. Meyer, B.J. (2010). Targeting X chromosomes for repression. Curr. Opin. Genet. Dev. *20*, 179–189.
9. Krasovec, M., Chester, M., Ridout, K., and Filatov, D.A. (2018). The mutation rate and the age of the sex chromosomes in *Silene latifolia*. Curr. Biol. *28*, 1832–1838.e4.
10. Chibalina, M.V., and Filatov, D.A. (2011). Plant Y chromosome degeneration is retarded by haploid purifying selection. Curr. Biol. *21*, 1475–1479.
11. Hough, J., Hollister, J.D., Wang, W., Barrett, S.C., and Wright, S.I. (2014). Genetic degeneration of old and young Y chromosomes in the flowering plant *Rumex hastatulus*. Proc. Natl. Acad. Sci. USA *111*, 7713–7718.
12. Muyle, A., Zemp, N., Deschamps, C., Mousset, S., Widmer, A., and Marais, G.A. (2012). Rapid *de novo* evolution of X chromosome dosage compensation in *Silene latifolia*, a plant with young sex chromosomes. PLoS Biol. *10*, e1001308.
13. Papadopulos, A.S., Chester, M., Ridout, K., and Filatov, D.A. (2015). Rapid Y degeneration and dosage compensation in plant sex chromosomes. Proc. Natl. Acad. Sci. USA *112*, 13021–13026.
14. Kazama, Y., Ishii, K., Aonuma, W., Ikeda, T., Kawamoto, H., Koizumi, A., Filatov, D.A., Chibalina, M., Bergero, R., Charlesworth, D., et al. (2016). A new physical mapping approach refines the sex-determining gene positions on the *Silene latifolia* Y-chromosome. Sci. Rep. *6*, 18917.
15. Mortazavi, A., Williams, B.A., McCue, K., Schaeffer, L., and Wold, B. (2008). Mapping and quantifying mammalian transcriptomes by RNA-seq. Nat. Methods *5*, 621–628.
16. Birchler, J.A. (2014). Facts and artifacts in studies of gene expression in aneuploids and sex chromosomes. Chromosoma *123*, 459–469.
17. Hou, J., Shi, X., Chen, C., Islam, M.S., Johnson, A.F., Kanno, T., Huettel, B., Yen, M.-R., Hsu, F.-M., Ji, T., et al. (2018). Global impacts of chromosomal imbalance on gene expression in *Arabidopsis* and other taxa. Proc. Natl. Acad. Sci. USA *115*, E11321–E11330.
18. Bergero, R., Qiu, S., Forrest, A., Borthwick, H., and Charlesworth, D. (2013). Expansion of the pseudo-autosomal region and ongoing

recombination suppression in the *Silene latifolia* sex chromosomes. Genetics *194*, 673–686.

19. Bergero, R., Qiu, S., and Charlesworth, D. (2015). Gene loss from a plant sex chromosome system. Curr. Biol. *25*, 1234–1240.
20. Sheltzer, J.M., Torres, E.M., Dunham, M.J., and Amon, A. (2012). Transcriptional consequences of aneuploidy. Proc. Natl. Acad. Sci. USA *109*, 12644–12649.
21. Malone, J.H., Cho, D.Y., Mattiuzzo, N.R., Artieri, C.G., Jiang, L., Dale, R.K., Smith, H.E., McDaniel, J., Munro, S., Salit, M., et al. (2012). Mediation of Drosophila autosomal dosage effects and compensation by network interactions. Genome Biol. *13*, r28.
22. Birchler, J.A., and Newton, K.J. (1981). Modulation of protein levels in chromosomal dosage series of maize: the biochemical basis of aneuploid syndromes. Genetics *99*, 247–266.
23. Veitia, R.A., Bottani, S., and Birchler, J.A. (2008). Cellular reactions to gene dosage imbalance: genomic, transcriptomic and proteomic effects. Trends Genet. *24*, 390–397.
24. Veitia, R.A. (2003). Nonlinear effects in macromolecular assembly and dosage sensitivity. J. Theor. Biol. *220*, 19–25.
25. Birchler, J.A. (2016). Parallel universes for models of X chromosome dosage compensation in Drosophila: a review. Cytogenet. Genome Res. *148*, 52–67.
26. Birchler, J.A. (1981). The genetic basis of dosage compensation of alcohol dehydrogenase-1 in maize. Genetics *97*, 625–637.
27. Birchler, J.A., Hiebert, J.C., and Paigen, K. (1990). Analysis of autosomal dosage compensation involving the alcohol dehydrogenase locus in *Drosophila melanogaster*. Genetics *124*, 679–686.
28. Birchler, J.A., and Veitia, R.A. (2012). Gene balance hypothesis: connecting issues of dosage sensitivity across biological disciplines. Proc. Natl. Acad. Sci. USA *109*, 14746–14753.
29. Sun, L., Johnson, A.F., Donohue, R.C., Li, J., Cheng, J., and Birchler, J.A. (2013). Dosage compensation and inverse effects in triple X metafemales of Drosophila. Proc. Natl. Acad. Sci. USA *110*, 7383–7388.
30. Torres, E.M., Sokolsky, T., Tucker, C.M., Chan, L.Y., Boselli, M., Dunham, M.J., and Amon, A. (2007). Effects of aneuploidy on cellular physiology and cell division in haploid yeast. Science *317*, 916–924.
31. Jansa, P., Homolka, D., Blatny, R., Mistrik, M., Bartek, J., and Forejt, J. (2014). Dosage compensation of an aneuploid genome in mouse spermatogenic cells. Biol. Reprod. *90*, 124.
32. Raznahan, A., Parikshak, N.N., Chandran, V., Blumenthal, J.D., Clasen, L.S., Alexander-Bloch, A.F., Zinn, A.R., Wangsa, D., Wise, J., Murphy, D.G.M., et al. (2018). Sex-chromosome dosage effects on gene expression in humans. Proc. Natl. Acad. Sci. USA *115*, 7398–7403.
33. Lucchesi, J.C., Kelly, W.G., and Panning, B. (2005). Chromatin remodeling in dosage compensation. Annu. Rev. Genet. *39*, 615–651.
34. Payer, B., and Lee, J.T. (2008). X chromosome dosage compensation: how mammals keep the balance. Annu. Rev. Genet. *42*, 733–772.
35. Strome, S., Kelly, W.G., Ercan, S., and Lieb, J.D. (2014). Regulation of the X chromosomes in Caenorhabditis elegans. Cold Spring Harb. Perspect. Biol. *6*, a018366.
36. Sharma, R., and Meister, P. (2016). Dosage compensation and nuclear organization: cluster to control chromosome-wide gene expression. Curr. Opin. Genet. Dev. *37*, 9–16.
37. Ferrari, F., Alekseyenko, A.A., Park, P.J., and Kuroda, M.I. (2014). Transcriptional control of a whole chromosome: emerging models for dosage compensation. Nat. Struct. Mol. Biol. *21*, 118–125.
38. Dong, P., Tu, X., Chu, P.Y., Lü, P., Zhu, N., Grierson, D., Du, B., Li, P., and Zhong, S. (2017). 3D chromatin architecture of large plant genomes determined by local A/B compartments. Mol. Plant *10*, 1497–1509.
39. Sotelo-Silveira, M., Chávez Montes, R.A., Sotelo-Silveira, J.R., Marsch-Martínez, N., and de Folter, S. (2018). Entering the next dimension: plant genomes in 3D. Trends Plant Sci. *23*, 598–612.
40. Kawashima, T., and Berger, F. (2014). Epigenetic reprogramming in plant sexual reproduction. Nat. Rev. Genet. *15*, 613–624.
41. Waters, S.A., Capraro, A., McIntyre, K.L., Marshall Graves, J.A., and Waters, P.D. (2018). The methylome of vertebrate sex chromosomes. Genes (Basel) *9*, 230.
42. Rodríguez Lorenzo, J.L., Hobza, R., and Vyskot, B. (2018). DNA methylation and genetic degeneration of the Y chromosome in the dioecious plant *Silene latifolia*. BMC Genomics *19*, 540.
43. Muyle, A., Zemp, N., Fruchard, C., Cegan, R., Vrana, J., Deschamps, C., Tavares, R., Hobza, R., Picard, F., Widmer, A., and Marais, G.A.B. (2018). Genomic imprinting mediates dosage compensation in a young plant XY system. Nat. Plants *4*, 677–680.
44. Li, H., and Durbin, R. (2009). Fast and accurate short read alignment with Burrows-Wheeler transform. Bioinformatics *25*, 1754–1760.
45. Li, H., Handsaker, B., Wysoker, A., Fennell, T., Ruan, J., Homer, N., Marth, G., Abecasis, G., and Durbin, R.; 1000 Genome Project Data Processing Subgroup (2009). The sequence alignment/map format and SAMtools. Bioinformatics *25*, 2078–2079.
46. Li, B., and Dewey, C.N. (2011). RSEM: accurate transcript quantification from RNA-Seq data with or without a reference genome. BMC Bioinformatics *12*, 323.
47. Langmead, B., and Salzberg, S.L. (2012). Fast gapped-read alignment with Bowtie 2. Nat. Methods *9*, 357–359.
48. McKenna, A., Hanna, M., Banks, E., Sivachenko, A., Cibulskis, K., Kernytsky, A., Garimella, K., Altshuler, D., Gabriel, S., Daly, M., and DePristo, M.A. (2010). The Genome Analysis Toolkit: a MapReduce framework for analyzing next-generation DNA sequencing data. Genome Res. *20*, 1297–1303.
49. Wu, T.D., Reeder, J., Lawrence, M., Becker, G., and Brauer, M.J. (2016). GMAP and GSNAP for genomic sequence alignment: enhancements to speed, accuracy, and functionality. Methods Mol. Biol. *1418*, 283–334.
50. Krueger, F., and Andrews, S.R. (2011). Bismark: a flexible aligner and methylation caller for Bisulfite-Seq applications. Bioinformatics *27*, 1571–1572.
51. R Development Core Team. (2014). R: A language and environment for statistical computing (R Foundation for Statistical Computing).
52. Kazama, Y., Sugiyama, R., Matsunaga, S., Shibata, F., Uchida, W., Hizume, M., and Kawano, S. (2003). Organization of the KpnI family of chromosomal distal-end satellite DNAs in *Silene latifolia*. J. Plant Res. *116*, 317–326.
53. Kazama, Y., Hirano, T., Saito, H., Liu, Y., Ohbu, S., Hayashi, Y., and Abe, T. (2011). Characterization of highly efficient heavy-ion mutagenesis in *Arabidopsis thaliana*. BMC Plant Biol. *11*, 161.
54. Li, P., Piao, Y., Shon, H.S., and Ryu, K.H. (2015). Comparing the normalization methods for the differential analysis of Illumina high-throughput RNA-seq data. BMC Bioinformatics *16*, 347.
55. Yang, Z. (2007). PAML 4: phylogenetic analysis by maximum likelihood. Mol. Biol. Evol. *24*, 1586–1591.
56. Ziller, M.J., Hansen, K.D., Meissner, A., and Aryee, M.J. (2015). Coverage recommendations for methylation analysis by whole-genome bisulfite sequencing. Nat. Methods *12*, 230–232, 1, 232.
57. Benjamini, Y., and Hochberg, Y. (1995). Controlling the false discovery rate: a practical and powerful approach to multiple testing. J. Roy. Stat. Soc. B Met. *57*, 289–300.

CellPress

STAR★METHODS

KEY RESOURCES TABLE

REAGENT OR RESOURCE	SOURCE	IDENTIFIER
CRITICAL COMMERCIAL ASSAYS		
Illumina paired end genomic sequencing	WTCHG, UK	Paired end genomic sequencing
Illumina transcriptome sequencing	WTCHG, UK	Paired end sequencing of polyA RNA
Illumina high throughput sequencing of bisulfite-treated genomic DNA	BGI, Hong Kong	Paired end bisulfite sequencing
QIAGEN RNeasy Plant Mini Kit	QIAGEN	cat # 74904
QIAGEN DNeasy Plant Kit	QIAGEN	cat # 69104
DEPOSITED DATA		
Genome sequencing data of *S. latifolia* deletion mutants EGP14, EGP17, mut3-98 and control (deposited 81Gb, 82Gb, 77Gb and 79Gb, respectively)	This paper	NCBI Bioproject: PRJNA474609
Bisulfite-treated genome sequence data of *S. latifolia* Y-deletion mutants ESS1, ESS4 and control (deposited 26Gb, 25Gb and 26Gb, respectively)	This paper	NCBI Bioproject: PRJNA474609
Transcriptomic data of 18 *S. latifolia* deletion mutants and controls (Data S1)	This paper	NCBI Bioproject: PRJNA474609
Transcriptomic data of parents and F2	[13]	NCBI Bioproject: PRJNA289919
EXPERIMENTAL MODELS: ORGANISMS/STRAINS		
S. latifolia deletion mutants	This paper	Listed in Table S1
SOFTWARE AND ALGORITHMS		
BWA v.0.7.12-r1039	[44]	http://bio-bwa.sourceforge.net/
Samtools v.1.2.1	[45]	http://www.htslib.org/doc/samtools.html
RSEM v.1.2.31	[46]	https://github.com/deweylab/RSEM
Bowtie2 v.2.1.0	[47]	http://bowtie-bio.sourceforge.net/bowtie2/index.shtml
GenomeAnalysisTK v.3.4-46	[48]	https://software.broadinstitute.org/gatk/
GSNAP v.2018-02-12	[49]	https://github.com/juliangehring/GMAP-GSNAP
Bismark v.0.20.1	[50]	https://www.bioinformatics.babraham.ac.uk/projects/bismark/
DelMapper	[14]	https://github.com/ion-beam-breeding/DelMapper
R v.3.4.4	[51]	https://www.r-project.org/

CONTACT FOR REAGENT AND RESOURCE SHARING

Further information regarding the manuscript and requests for reagents may be directed to, and will be fulfilled by the Lead Contact, Dmitry A. Filatov (Dmitry.Filatov@plants.ox.ac.uk).

EXPERIMENTAL MODEL AND SUBJECT DETAILS

In this study we used mutant and wild-type *Silene latifolia* plants all of which belonged to the same highly inbred K-line that was generated by full-sib mating for 14 generations by Kazama and colleagues [52]. The draft genome sequence of that inbred line was published previously [13]. The 101 Y-deletion mutants used for deletion mapping of the Y chromosome and the phenotypes of these mutants are listed in Table S1. All plants used in the study were grown in the glasshouse at ambient temperature with long day (16h) lighting regime.

METHOD DETAILS

Deletion mutants

All the mutants that were used in this study were generated using the methodology described previously [14]. Briefly, pollen grains or dry seeds of the inbred K-line were irradiated with carbon ion beams with a linear energy transfer (LET) of 30 keV μm^{-1} or iron ion beams with a LET of 640 keV μm^{-1} to generate random deletions. The tissue and dose of irradiation used for each mutant are listed in Table S1. Heavy-ion beams have high LET and thus are expected to induce more localized deletions than low-LET radiation, such a X rays or γ rays [53]. The mutants were found by changes in the floral phenotypes observed in the M1 or M2 generations. The deletions were identified and verified by PCR and sequencing. The PCR primers used for this purpose are listed in Table S2.

A subset of the mutants used for Y-deletion mapping for which plants were still alive and actively growing at the time of the study, were used for high throughput sequencing (Table 1). The Y-deletion mutants sequenced in this study can be divided into groups according to the deletion map on the Y chromosome and the phenotypic effect of the deletion (Figure 2A and Table S1). The first group comprised hermaphroditic mutants (m441, EGP4, EGP14, EGP15, EGP16 and EGP17) which lost the gynoecium suppressing function (GSF) gene and a number of adjacent genes. The second group includes asexual mutants (K034, ESS1, ESS4, ESS5, ESS7, ESS8, ESS10 and ESS11) which lost the stamen promoting function (SPF) gene and adjacent genes. One of these asexual mutants, ESS10 also contained a large deletion in the q arm of the Y chromosome, which allowed us to test whether the deletions in the q arm of the Y chromosome trigger IDC. In addition to that, we analyzed a multiple deletion mutant (GPSS1) which lost both GSF and SPF and their adjacent genes and two Y-deletion mutants with normal male phenotype (M51 and mut3-70) that contained deletions in the q arm of the Y chromosome.

Deletion mapping of the Y chromosome

Construction of the Y-deletion map for the *S. latifolia* inbred K-line followed the same methodology as described previously [14]. Briefly, 163 STS markers were used to determine the location of Y deletions in the mutants (Tables S1 and S2). The resulting marker presence/absence data was input into DelMapper program [14] with the "Any" option and the option to specify the number of the marker clusters as 14. Under these options, the markers were clustered into 14 clusters and the markers in each cluster were summarized in a virtual marker. DelMapper was used to calculate the most plausible order(s) of the virtual markers. In each cluster, DelMapper calculated the most plausible order(s) of the markers recursively. In the calculations, the ends of the mapping region were assumed to have same deletion status (present or absent) as the adjacent marker.

Genomic sequencing

To facilitate the identification and verification of deleted genes we sequenced the genomes of three deletion mutants (EGP14, EGP17 and mut3-98) and of a non-irradiated male plant of the K-line. In addition to that, we used genome sequence data from non-irradiated plants of the inbred K-line that were published previously [13]. DNA for genomic sequencing was extracted from fresh leaves using the DNeasy Plant Mini kit (QIAGEN). For high-throughput sequencing, PCR-free Illumina libraries were prepared at Oxford Wellcome Trust genomic center (WTCHG). These libraries were sequenced on HiSeq2500 instrument at WTCHG. Reads were aligned against the reference [13] with BWA v.0.7.12 (bwa aln, standard parameters except -n 1) [44] and the read alignment files were processed with Samtools v.1.2 [45] to remove duplicate reads (with mapping quality threshold = 20) and obtain the coverage for different regions and genes across the genome (using Samtools idxstats).

Transcriptome sequencing

Mutant and control plants used for transcriptome sequencing were grown in the glasshouse with standard long day (16h light) conditions. RNA was extracted from fresh leaves and flower buds with the RNeasy Plant Mini Kit (QIAGEN), including the DNase treatment step recommended by the QIAGEN manual for that kit. Transcriptome sequencing libraries were prepared with Illumina TruSeq mRNA Library Prep Kit at the WTCHG. The sequencing of the libraries was conducted at the WTCHG on Illumina HiSeq4000 instrument with 75 bp paired-ends reads. Up to three technical replicates per plant were extracted at different times and sequenced separately to minimize expression noise (Data S1). Reads were aligned against the reference transcriptome [13] with Bowtie2 v.2.1.0 with standard parameters (-n 2 -l 25 -I 1 -X 1000 -p 1 -a -m 200) and expression values were obtained for each gene using RSEM v.1.2.31 [46] with default parameters to extract the FPKM values.

Identification of deleted genes for expression analyses

A previous study [14] identified and verified 71 loci deleted in the Y-deletion mutants; 35 of these genes were used in our expression analyses. The rest of the previously verified Y-linked deletions could not be used due to unavailability of plant material for these mutants. Using genomic sequence data for mutant males (EGP14 and EGP17) and non-irradiated controls we double-checked the Y deletions identified previously [14]. As expected, the Y gametologs had zero coverage in the deletion mutants. The gene expression level was used as another validation of the deleted Y-linked genes, which confirmed zero coverage for previously verified Y-linked genes in the deletion mutants, while the X-copy (in mutants and non mutants) and the Y-copy in non mutants were expressed (Data S1). By extension, we identified new Y deletions if (*i*) the Y gametolog had a coverage of zero and (*ii*) the X gametolog was expressed in at least a subset of mutants, and if (*iii*) both X and Y gametologs were expressed in non mutant individuals.

The pollen grain nuclei are haploid, so a deletion of a Y-linked copy of a sex-lined gene due to pollen irradiation leads to a single maternal X-linked gametolog present in the adult male progeny. Similarly, for autosomal deletions the mutant is heterozygous for the deletion and has a single copy of the genes within the deleted region. This allowed us to use a sequence coverage approach to detect autosomal deletions, where the mutants are expected to have half the coverage of the non mutants, using the RPKM value (Table S3). The RPKM value was obtained for each gene by taking the number of mapped reads divided by the length of the gene and the total million number of mapped reads.

Expression analysis of deletion mutants

Expression was quantified with the commonly used measure FPKM (fragments per kilobase per million mapped fragments) value that combines inter- and intra-sample normalization by rescaling read counts per gene to correct for differences in both library size and gene length [15]. This approach was shown to be one of the most accurate ways to normalize RNA-seq data [54]. FPKM values show high correlation between the technical replicates (Pearson correlation, *rho* > 0.82, P value < 2.2×10^{-16}), indicating high quality of data acquisition. The average FPKM values across technical replicates for each individual were used for further analyses.

The Y and X gametologs in *S. latifolia* diverged 6 to 11 million years ago, as was recently revealed by direct estimation of the spontaneous mutation rate in this species [9]. As such, the X- and Y-linked gene copies are sufficiently divergent to accurately estimate expression of each copy separately. In particular, as demonstrated in our previous study [13], the Y-linked gametologs show zero expression in females, which indicates no cross-mapping of X reads to Y-linked gametologs. Furthermore, the presence or absence of Y gametologs in the reference sequence during read mapping does not alter the measurements of expression of X-linked gametologs.

The sex-linkage of genes used in the analyses was established in our previous work [10, 13]. We defined X_{del} and X_{nondel} as the level of expression of the X gametologs with deleted and non-deleted Y gametologs, respectively, in the deletion mutant, while X_c denotes the expression of the X-linked genes in the controls. We compared the expression level of the X gametolog between mutants (X_{del} or X_{nondel}) and control plants (X_c), calculating the X_{del}/X_c or X_{nondel}/X_c ratios for each gene. Sex-linked genes were divided into different categories depending on divergence between homologous X- and Y-linked gene copies (Figures S1A–S1G), the extent of Y degeneration (Figures S1I–S1O), and the position of Y-linked gene in the map of the Y chromosome (Figures 2 and S2). This enabled us to test whether IDC is more pronounced in any of these categories. The divergence between homologous X- and Y-linked gene copies was calculated at synonymous positions (*dS*) using paml [55]. According to *dS* between X- and Y-linked gametologs, sex-linked genes were split into groups with low (*dS*<0.04), intermediate (0.04 < *dS*<0.08) and relatively high (*dS*>0.08) divergence. The degeneration of the Y-linked gametologs was estimated according to the extent of expression difference between the X and Y gametologs in the controls (Y_c/X_c); (*i*) genes with a Y_c/X_c ratio below 0.3 were considered to have highly degenerate Y gametologs; (*ii*) genes with a Y_c/X_c ratio between 0.3 and 0.7 were considered to have partially degenerated Y gametologs; (*iii*) genes with a Y_c/X_c ratio above 0.7 were treated as having non-degenerate Y gametologs.

Methylation analysis

In order to test whether methylation has a role in upregulation of X_{del} genes in *S. latifolia*, we analyzed genomic sequences of bisulfite-treated DNA of two mutants ESS1 and ESS4 and the non-irradiated control. DNA was extracted from leaves with the DNA plant easy kit following standard instructions. Bisulfite treatment, library construction and high-throughput sequencing (150b paired end) on Illumina Novaseq S4 platform was done by Beijing genomic institute. The resulting sequence reads have been aligned against the reference transcriptome and reference genome with Bismark [50] using standard parameters (–comprehensive option). The analysis of methylation was conducted in coding sequences and 5′ regions (upstream 5kb) of the X_{del}, X_{nondel} and autosomal genes used in the study. The global methylation state of CpG, CHH and CHG sites have been extracted with Bismark for autosomal and sex-linked genes with an average coverage $\geq$ 3. As recommended for such analyses [56], methylation was analyzed only at the positions with minimal coverage of 5. We used the methylation percentage ratio between the mutants and the control to detect a variation in methylation state. The significance of difference was tested with Wilcoxon rank sum test.

Genomic imprinting analysis

In order to test whether imprinting is involved in *S. latifolia* dosage compensation, we followed the approach of Muyle et al. [43], but used an independent dataset published previously [13]. The dataset comprised transcriptome data for parents and 52 progeny (20 males and 32 females) of a *S. latifolia* genetic cross [13]. High coverage genome sequence data for parents and grand-parents of that genetic cross were also available [9], which helped to establish the genotypes of alleles segregating in the expressed genes. The sequence reads were mapped against the reference transcriptome [13] using GSNAP [49] with the allele tolerance option (-v) that allows a second reference allele for a site. This avoids any reference allele mapping bias using previously identified SNP as a second reference [13]. The resulting bam files were processed with Samtools [45] and Picardtools (http://broadinstitute.github.io/picard) for a new round of SNP calling using HaplotypeCaller from GATK [48] to get the coverage of each allele of previously identified polymorphic positions. The RPKM values were calculated at each polymorphic position and then the average of allelic expression (*E*) was calculated for each gene in each individual with the same formula that was used by Muyle et al. [43]: $E = r/(n*l)$, where r is the total number of reads covering one of the two alleles (paternal and maternal) of a gene, n is the number of SNP positions in the gene and l the library size. The E values are calculated separately for the alleles of maternal and paternal origin ($E_{maternal}$ and $E_{paternal}$, respectively) for each gene in each individual. Then, we averaged all E values of the same origin across all the progeny of the same sex,

which yielded four separate E-values per gene: $E_{maternal\_in\_females}$, $E_{paternal\_in\_females}$, $E_{maternal\_in\_males}$ and $E_{paternal\_in\_males}$. The distributions of $E_{maternal\_in\_females}$ / $E_{paternal\_in\_females}$ and $E_{maternal\_in\_males}$ / $E_{paternal\_in\_males}$ ratios were analyzed separately for X-linked and autosomal genes (Figure S4). Furthermore, following Muyle et al. [43], sex-linked genes were divided into three categories (Y/X > 0.75; 0.75 > Y/X > 0.25; Y/X < 0.25) according to the extent Y degeneration and the relative expression of Y versus X gametologs (Y/X). The expression of the X and Y gametologs was measured previously [13].

QUANTIFICATION AND STATISTICAL ANALYSIS

Throughout the paper the significance of expression difference between the categories was tested using the two-sided Wilcoxon rank sum test. Relative expression (mutants/controls expression ratios) for each category was summarized as probability density histograms (with kernel smoothing function), as well as in the form of notched box-and-whisker plots generated in R. In the box-and-whisker plots, the box spans the first quartile (Q1) to the third quartile (Q3); the line inside the box shows the median; the notches represent the 95% confidence interval for the median; wiskers show the range between Q1 - 1.5x(Q3 - Q1) and Q3 + 1.5x(Q3 - Q1) and should include 99.3% of the data if it were normally distributed. All the boxplots show the outliers represented with small circles outside the range of the wiskers. The number inside the box lists the number of genes analyzed for each category. Differential expression was tested for every gene with t test comparing expression (FPKM) across the technical replicates for the controls and the particular mutant. The p values of the t tests were corrected for multiple testing using R function *p.adjust* with Benjamini and Hochberg false discovery rate (FDR) correction [57]. The principle components analysis of expression was conducted using R function *prcomp* and the PCA plot (Figure 2B) was generated with *ggbiplot* function from R package *ggbiplot* v0.55. Volcano plots of X_{del}, X_{nondel} and autosomal genes (Figure S2) were generated using the *volcanoPlot* function in R v3.5.1. In these plots the significance of change in expression between the mutants and controls (y axis) was plotted against log2-transformed extent of expression change (x axis) for each gene. The significance of the expression changes between mutants and controls were tested with *t.test* in R.

DATA AND SOFTWARE AVAILABILITY

All genomic and transcriptomic raw sequence reads we used are available from NCBI under the Bioprojects PRJNA474609 and PRJNA289919.

Current Biology, Volume *29*

Supplemental Information

Immediate Dosage Compensation
Is Triggered by the Deletion
of Y-Linked Genes in *Silene latifolia*

Marc Krasovec, Yusuke Kazama, Kotaro Ishii, Tomoko Abe, and Dmitry A. Filatov

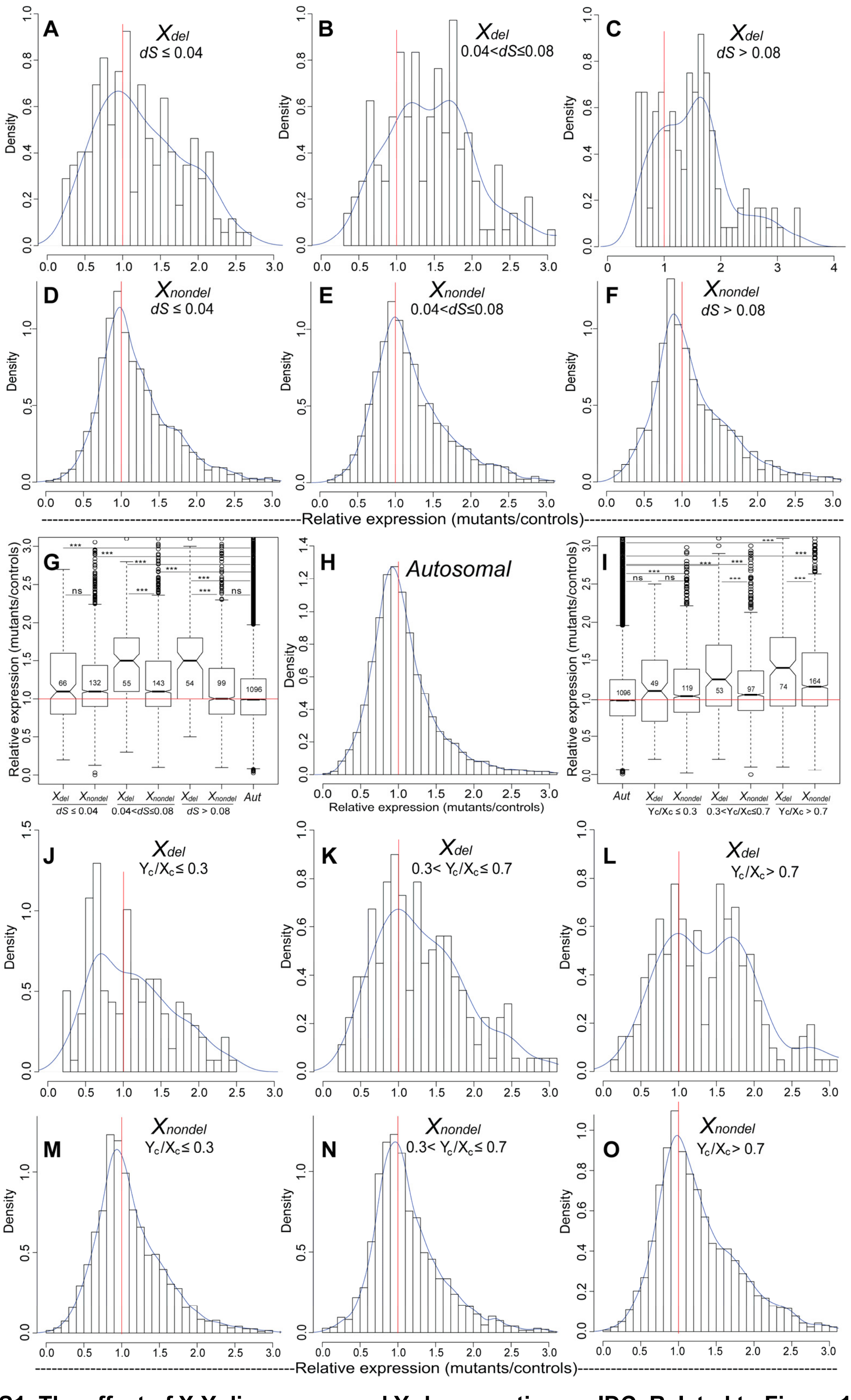

Figure S1. The effect of X:Y divergence and Y-degeneration on IDC. Related to Figure 1. Up-regulation of X_{del} and X_{nondel} gene expression in the deletion mutants compared to non-irradiated controls depending on synonymous divergence (*dS*) between X- and Y-linked gametologs (panels A to G) and the extent of degeneration of their Y-linked gametologs (panels I to O). The extent of Y-degeneration is measured as Y/X expression ratio in controls (Y_c/X_c). Significance of difference between different categories (Wilcoxon rank sum test, *** $P < 0.001$) and numbers of genes analysed are shown in panels G and I.

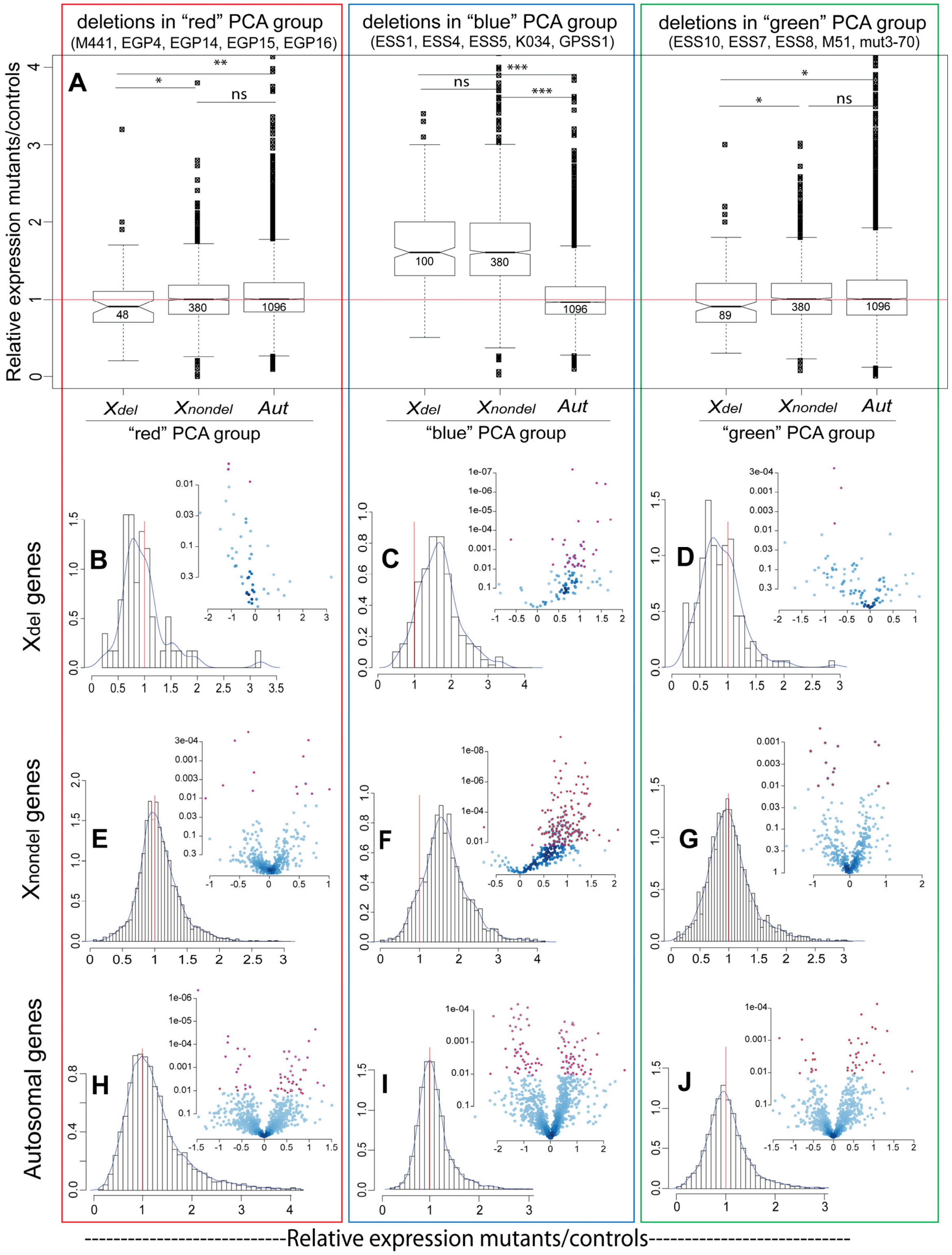

Figure S2. **Up-regulation of X-linked genes is caused by deletions in the mutants of the "blue" PCA cluster**. **Related to Figure 2**.This figure shows the same analyses as in Figure 2C to 2L but with normalisation by non-irradiated mutants. The number of genes in each category is shown within the boxes (panel A). Significance of difference between different gene categories was tested with Wilcoxon rank sum test: * *P*<0.05; ** *P*<0.01; *** *P*<0.001. The insets for each histogram (panels B to J) show volcano plots of significance of expression change (vertical axis) versus log_2-fold change (horizontal axis) in gene expression in mutants relative to controls. The genes showing significant (t-test, *P*<0.01) expression change in mutants compared to controls are highlighted in red.

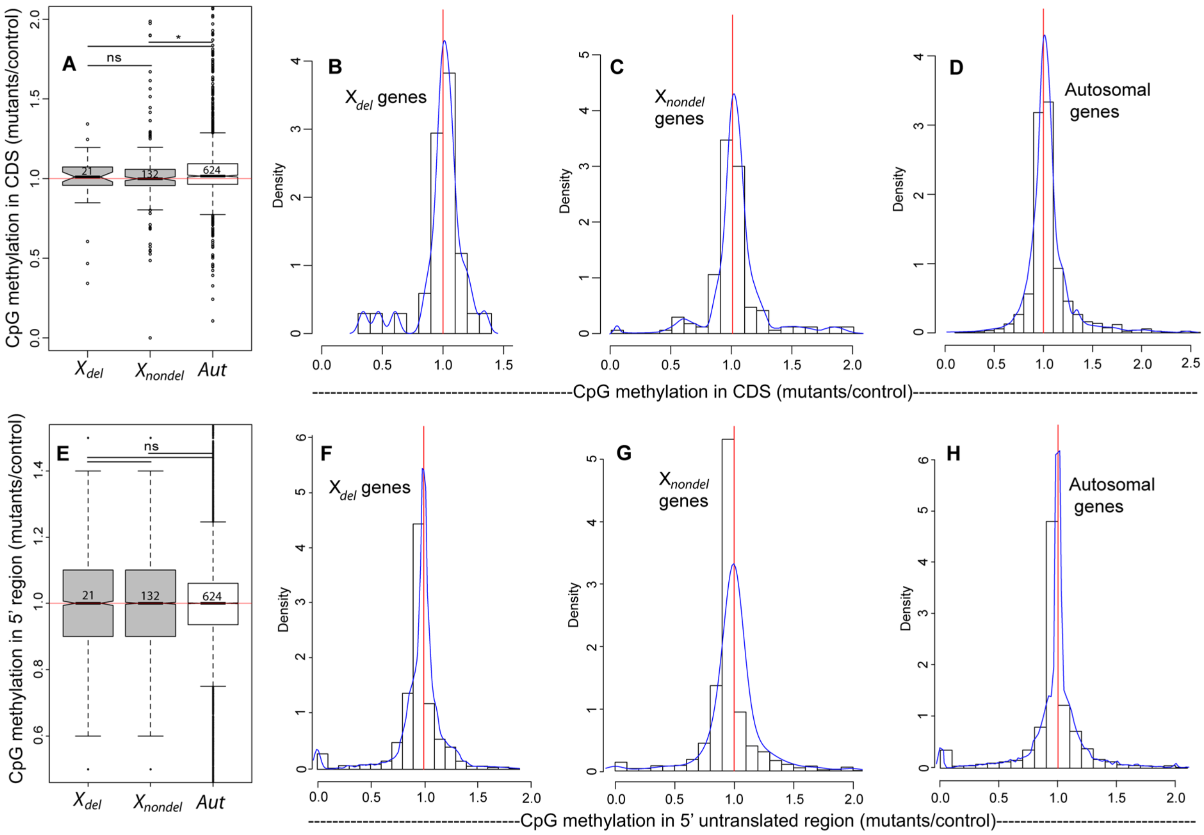

Figure S3. DNA methylation is not involved in IDC. **Related to Figure 2**. DNA methylation in leaves of mutants ESS1 and ESS4 relative to control at CpG sites in coding regions (A to D) and 5' untranslated regions (E to H) of X_{del}, X_{nondel} and autosomal (*Aut*) genes. The red lines in all panels shows the null expectation for no difference between the mutants and control. The blue curve in panels B to D and F to H show the kernel smoothing function. The number of genes analysed in each category is shown within the boxes on the boxplots. Significance of difference between different gene categories was tested with Wilcoxon rank sum test: * P<0.05; ns, non-significant.

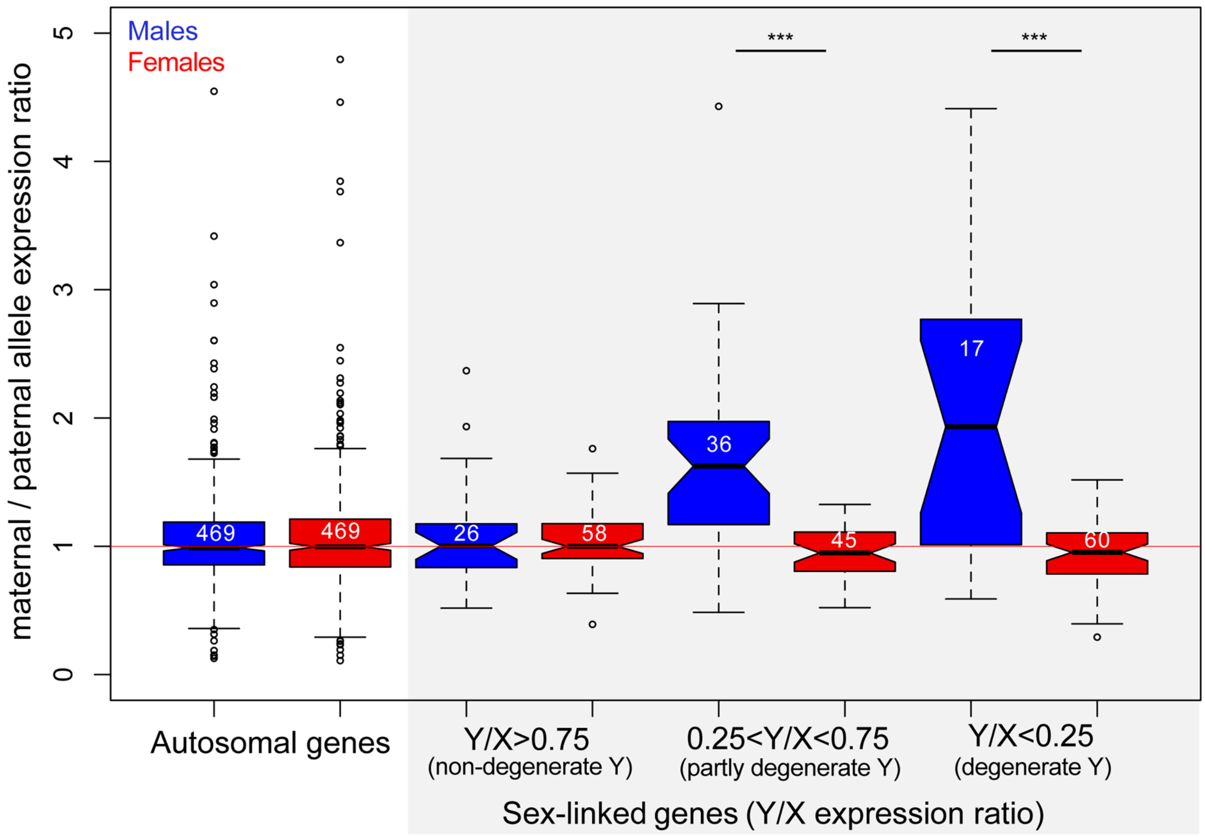

Figure S4. No evidence for maternal imprinting in *S. latifolia*. Related to Figure 2. Transcriptome sequence data from a genetic cross, including parents and 52 progeny (20 males and 32 females) was used to track the inheritance of maternal and paternal alleles and to estimate allele-specific expression as described in the methods. The expression ratios of maternal and paternal alleles in females (red) do not differ significantly from the expectation of equal expression. In males (blue), the deviation from equal expression in two categories of sex-linked genes is caused by Y-degeneration that results in weaker expression of Y-gametologs (paternal) compared to their X-gametologs (maternal) in males. Number of genes analysed in each category is shown inside the boxes on the boxplot. Wilcoxon rank sum test: *** P < 0.001.

SUPPLEMENTAL REFERENCE

S1. Kazama, Y., Ishii, K., Aonuma, W., Ikeda, T., Kawamoto, H., Koizumi, A., Filatov, D.A., Chibalina, M., Bergero, R., Charlesworth, D., et al. (2016). A new physical mapping approach refines the sex-determining gene positions on the *Silene latifolia* Y-chromosome. Sci Rep *6*, 18917.
